# Molecular basis of differential adventitious rooting competence in poplar genotypes

**DOI:** 10.1093/jxb/erac126

**Published:** 2022-03-24

**Authors:** Alok Ranjan, Irene Perrone, Sanaria Alallaq, Rajesh Singh, Adeline Rigal, Federica Brunoni, Walter Chitarra, Frederic Guinet, Annegret Kohler, Francis Martin, Nathaniel R Street, Rishikesh Bhalerao, Valérie Legué, Catherine Bellini

**Affiliations:** Umeå Plant Science Centre, Department of Plant Physiology, Umeå University, SE-90736 Umeå, Sweden; Umeå Plant Science Centre, Department of Plant Physiology, Umeå University, SE-90736 Umeå, Sweden; Institute for Sustainable Plant Protection, National Research Council of Italy (IPSP-CNR), I-10135 Torino, Italy; Umeå Plant Science Centre, Department of Plant Physiology, Umeå University, SE-90736 Umeå, Sweden; Department of Biology, College of Science for Women, Baghdad University,10071, Baghdad, Iraq; Umeå Plant Science Centre, Department of Forest Genetics and Physiology, Swedish Agricultural University, SE-90183 Umeå, Sweden; Université de Lorraine, INRAE, UMR Interactions Arbres/Microorganismes, Laboratory of Excellence ARBRE, INRAE GrandEst-Nancy, Champenoux, 54280France; Umeå Plant Science Centre, Department of Plant Physiology, Umeå University, SE-90736 Umeå, Sweden; Institute for Sustainable Plant Protection, National Research Council of Italy (IPSP-CNR), I-10135 Torino, Italy; Research Centre for Viticulture and Enology, Council for Agricultural Research and Economics (CREA-VE), I-31015 Conegliano (TV), Italy; Université de Lorraine, INRAE, UMR Interactions Arbres/Microorganismes, Laboratory of Excellence ARBRE, INRAE GrandEst-Nancy, Champenoux, 54280France; Université de Lorraine, INRAE, UMR Interactions Arbres/Microorganismes, Laboratory of Excellence ARBRE, INRAE GrandEst-Nancy, Champenoux, 54280France; Université de Lorraine, INRAE, UMR Interactions Arbres/Microorganismes, Laboratory of Excellence ARBRE, INRAE GrandEst-Nancy, Champenoux, 54280France; Umeå Plant Science Centre, Department of Plant Physiology, Umeå University, SE-90736 Umeå, Sweden; Umeå Plant Science Centre, Department of Forest Genetics and Physiology, Swedish Agricultural University, SE-90183 Umeå, Sweden; Université de Lorraine, INRAE, UMR Interactions Arbres/Microorganismes, Laboratory of Excellence ARBRE, INRAE GrandEst-Nancy, Champenoux, 54280France; Umeå Plant Science Centre, Department of Plant Physiology, Umeå University, SE-90736 Umeå, Sweden; Université Paris-Saclay, INRAE, AgroParisTech, Institut Jean-Pierre Bourgin (IJPB), 78000, Versailles, France; Nanjing Agricultural University, China

**Keywords:** Adventitious roots, cambium, hybrid aspen, hybrid poplar, *Populus* spp, stem cuttings

## Abstract

Recalcitrant adventitious root (AR) development is a major hurdle in propagating commercially important woody plants. Although significant progress has been made to identify genes involved in subsequent steps of AR development, the molecular basis of differences in apparent recalcitrance to form AR between easy-to-root and difficult-to-root genotypes remains unknown. To address this, we generated cambium tissue-specific transcriptomic data from stem cuttings of hybrid aspen, T89 (difficult-to-root) and hybrid poplar OP42 (easy-to-root), and used transgenic approaches to verify the role of several transcription factors in the control of adventitious rooting. Increased peroxidase activity was positively correlated with better rooting. We found differentially expressed genes encoding reactive oxygen species scavenging proteins to be enriched in OP42 compared with T89. A greater number of differentially expressed transcription factors in cambium cells of OP42 compared with T89 was revealed by a more intense transcriptional reprograming in the former. *PtMYC2*, a potential negative regulator, was less expressed in OP42 compared with T89. Using transgenic approaches, we demonstrated that *PttARF17.1* and *PttMYC2.1* negatively regulate adventitious rooting. Our results provide insights into the molecular basis of genotypic differences in AR and implicate differential expression of the master regulator MYC2 as a critical player in this process.

## Teaser

Comparative transcriptomic analysis from the cambium of two *Populus*genotypes with contrasting adventitious rooting phenotype reveals higher transcriptional activity in the easy-to-root compared with the difficult-to-root genotype.

## Introduction

In the 1990s, only 3% of the world’s forested land were plantations for wood production. However, despite this small percentage, it still provided more than one third of total industrial wood production (Kirilenko and Sedjo, 2007). The shift of production from natural forests to plantations is projected to accelerate and is expected to rise to 75% in the 2050s (Kirilenko and Sedjo, 2007). Operating plantations is expensive and requires high productivity per hectare, which in turn requires good quality, i.e. genetically improved planting stock. Many forest companies are therefore currently considering clonal propagation in addition to, or in conjunction with, their breeding programmes. This aims to propagate elite genotypes from available genetic diversity and maximise the productivity of selected high-value hybrid clones (Bozzano *et al.*, 2014). The genus *Populus* comprises about 30 species; its wood forms an abundant and renewable source of biomaterials and bioenergy (Ragauskas *et al.*, 2006). The propagation of poplar species depends primarily on adventitious root (AR) formation from detached stem cuttings (Dickmann, 2006), but one major constraint for vegetative propagation of some economically important elite genotypes is incompetence or rapid loss of capacity in forming AR (Bellini *et al.*, 2014; Brunoni *et al.*, 2019; Bannoud and Bellini, 2021). AR development is a complex, heritable trait controlled by many endogenous regulatory factors, and quite influenced by environmental factors (Bellini *et al.*, 2014; Bannoud and Bellini, 2021). The rooting capacity of cuttings varies among individuals within species, populations, or even clones (Abarca and Díaz-Sala, 2009a, 2009b). Few studies have reported the genetic variability of AR development of *Populus* hardwood cuttings. Zhang *et al.* (2009) reported quantitative trait loci (QTL) that control two AR growth parameters in a full-sib family of 93 hybrids, derived from an interspecific cross between two *Populus* species, *P. deltoides* and *P. euramericana*, which are defined as difficult-to-root and easy-to-root, respectively. They showed that the maximum root length and the total AR number correlated and were under strong genetic control, which supports earlier QTL analysis performed on forest trees (reviewed in Geiss *et al.*, 2009). Several studies focusing on AR development in poplar have identified a number of genes involved in its regulation (Ramirez-Carvajal *et al.*, 2009; Rigal *et al.*, 2012; Trupiano *et al.*, 2013; Wuddineh *et al.*, 2015; Xu *et al.*, 2015; Yordanov *et al.*, 2017; Li *et al.*, 2018; Liu *et al.*, 2020; Wang *et al.*, 2020; Wei *et al.*, 2020; Yue *et al.*, 2020; Zhang *et al.*, 2020; Xu *et al.*, 2021) including large-scale data analyses identifying regulators (Ribeiro *et al.*, 2016; Zhang *et al.*, 2019) and pharmacological assays of physiological regulators (Gou *et al.*, 2010; Mauriat *et al.*, 2014; Zhang *et al.*, 2019).

All these studies resulted in a substantial increase in our understanding of the molecular mechanisms that control successive steps of AR development, but the molecular differences in recalcitrance to form AR between easy-to-root and difficult-to-root genotypes remains unknown. To address this question, we compared the transcriptome of cambium cells obtained immediately after cutting and 24 h later, by laser capture microdissection (LCM) from *P. trichocarpa* × *P. maximowiczii* (clone OP42) which we defined as ‘easy-to-root from woody stem cuttings’, and the hybrid aspen *P. tremula* × *P. tremuloides* (clone T89) which we defined as ‘difficult-to-root from woody stem cuttings’. OP42 is one of the poplar clones used most widely, both in Northern Europe and worldwide (Taeroe *et al.*, 2015). It can easily be propagated from dormant stem cuttings. In contrast, the hybrid aspen T89 cannot be propagated *via* dormant stem cuttings but can be easily propagated *in vitro,* and is very amenable to genetic transformation (Nilsson *et al.*, 1992). The analysis of the transcriptomic dataset showed more differentially expressed genes encoding transcription factors (TFs) in OP42 than in T89. We identified several TFs that could explain differences in ability to produce adventitious roots. We showed that up-regulation of the jasmonate (JA) signalling pathway in the cambium of T89 could be one cause of the failure to produce adventitious roots.

## Materials and methods

### Plant growth conditions and rooting assays

The hybrid aspen (*P. tremula L*. × *P. tremuloides Michx*), clone T89, and the hybrid poplar (*P. trichocarpa × P. maximowiczii*) clone OP42, were propagated *in vitro* for 4 weeks as described in Karlberg *et al.* (2011) and shown in Supplementary Fig. S1A. More precisely, plants were grown in plastic jars containing sterile half-strength Murashige and Skoog medium (Duchefa, The Netherlands), pH 5.6, at an average temperature of 25 ± 1 °C, under an 18 h/6 h light/dark cycle. Light at 150 μmol m^-2^ s^-1^ was provided by warm white fluorescent tubes. For *in vitro* rooting assays, 3 cm cuttings with four to five leaves in the case of T89, and two to three leaves in the case of *P. trichocarpa × P. maximowiczii* clone OP42 plantlets, were collected and transferred into smaller rectangular jars containing fresh sterile medium, as above (Supplementary Fig. S1B, D). The number of ARs was scored from day five after cutting, until day 14. Three replicates of 15 stem cuttings each were analysed. For the jasmonic acid and auxin treatments, cuttings from 4-week-old *in vitro* grown T89 and OP42 plantlets were transferred to fresh sterile medium with or without methyl jasmonate (MeJA) at 5 μM, 10 μM, or 20 μM, or with or without indole acetic acid (IAA) at 0.1 nM or 10 nM.

For the rooting assay in hydroponic conditions, *in vitro* plants of hybrid aspen (*P. tremula L*. × *P. tremuloides Michx*), clone T89, and hybrid poplar (*P. trichocarpa× P. maximowiczii*) clone OP42, that had been propagated *in vitro* for 4 weeks were transferred to soil and kept in the greenhouse for three months (16 h light, 21°C; 8 h dark 18 °C). After 3 months, 20 cm long stem cuttings were taken from the third internode below the shoot apex from T89 and OP42 plants. After removal of all leaves and buds except for the higher axillary bud (Supplementary Fig. S1C, E), the cuttings were transferred to hydroponic conditions in the greenhouse. The nutrient solution was composed of a modified Hoagland solution, as described in Plett *et al.* (2011). Photos of the ARs were taken using a Canon EOS 350 digital camera and Discovery V.8 stereomicroscope fitted with a Zeiss camera (Zeiss, Sweden; Supplementary Fig. S1C, E).

### Histological analysis of stem cuttings *in vitro*

For histological analysis of stems, 5 mm stem fragments were taken at the base of cuttings 4 or 5 d after cutting. Samples were vacuum infiltrated with a fixation medium (10 ml of 37% formaldehyde, 5 ml of 5% acetic acid, 50 ml of 100% ethanol and 35 ml of water) for 20 s and left for 24 h at 20 °C. The samples were then washed in 70% ethanol for 10 min and transferred into fresh 70% ethanol until required for use. Samples were then gradually dehydrated in an ethanol series (80%, 90%, 96% for 2 h each, and 100% overnight at 20 °C). The 100% ethanol was gradually replaced by HistoChoice tissue fixative (VWR Life, Sweden) in three steps of 1:3, 1:1, 3:1 (EtOH: HistoChoice), then with pure HistoChoice twice in 1 h. The HistoChoice fixative was gradually replaced with Paraplast Plus for tissue embedding (Sigma-Aldrich, USA) over 6 d. Ten µm cross or longitudinal sections were made with a rotary microtome (Zeiss, Germany) and stained with safranin and alcian blue (Sigma-Aldrich, USA) in a ratio of 1:2; using methods from Hamann *et al.* (2011). Stem sections taken from cuttings in hydroponic conditions were obtained using a vibratome (Leica Biosystems, UK). Following this, 20 μm sections were stained as described above.

### Tissue preparation before laser capture microdissection

#### Sampling, fixation, and cryoprotection steps

The basal 5 mm stem pieces of T89 and OP42 cuttings were harvested immediately after excision from greenhouse-grown plants (time T_0_) and after 24 h of hydroponic culture (time T_1_; Supplementary Fig. S2A-C). Three biological replicates of tissue samples were collected at each time point (T_0_ and T_1_) from both OP42 and T89 (12 samples in total = three biological replicates × two genotypes × two time points). Immediately after the sampling, stem pieces were split in half longitudinally and subjected to fixation and cryoprotection steps before the laser microdissection. We used the protocol described at https://schnablelab.plantgenomics.iastate.edu/resources/protocols/, slightly modified as follows: samples were soaked in cold ethanol-acetic acid (EAA) Farmer’s fixative solution, containing 75% (v/v) ethanol and 25% (v/v) acetic acid, and vacuum infiltrated on ice at 400 mm Hg for 20 min. After 1 h incubation at 4 °C, another step of vacuum infiltration with fresh Farmer`s solution was performed (400 mm Hg for 20 min). Samples were then kept at 4 °C overnight. The following day, the fixative solution was removed and the samples transferred into a 10% sucrose solution prepared with 1× phosphate buffered saline (PBS, 137 mM NaCl, 8 mM Na_2_PO_4_, 2.68 mM KCl, 1.47 mM KH_2_PO_4_), vacuum infiltrated on ice at 400 mm Hg for 15 min. Samples were left incubating for 1 h at 4 °C, then vacuum infiltrated with a 15% sucrose solution (400 mm Hg for 15 min). Samples were then incubated overnight at 4 °C; then frozen in liquid nitrogen and stored at –80 °C until cryosectioning.

### Cryosectioning

The day before cryosectioning, membrane slides for laser microdissection (FrameSlide PET, Zeiss; Fisher Scientific, UK) were treated with RNaseZap (Sigma-Aldrich, USA), rinsed twice with diethylpyrocarbonate (DEPC) water and dried for 2 h at 37 °C. Immediately before sectioning, slides were further treated with UV light for 30 min to improve adhesion of sections. Tweezers and a cryostat knife were sterilised at 180 °C for 4 h. The chamber temperature of the cryostat (Leica CM1850, Germany) was set at –25 °C. The instruments including tweezers, knives, and polyethylene teraphthalate (PET)-membrane coated slides were transferred into the chamber 20 min before sectioning. Samples were transferred from a –80 °C freezer to the cryostat in liquid nitrogen. They were fixed with Tissue-Tek® optimal cutting temperature (OCT) compound onto a specimen stage directly in the cryochamber. To avoid embedding and the presence of OCT compound on membrane slides, stem segments were mounted to allow cambium collection from tangential cryosections (Supplementary Fig. S2D). Sections of 25 µm were transferred with tweezers onto membrane slides, then moved to a Petri dish at 20 °C. Sections were then treated with 70% ethanol for 5 min at room temperature, followed by 95% ethanol for 2 min on ice, and 100% ethanol for 2 min on ice. In these dehydration steps, ethanol was applied and removed directly onto the membrane slide chamber with a sterile plastic Pasteur pipette, being careful not to damage the membrane. After ethanol removal, sections were air-dried for 5 min before being cut at the microdissector (Zeiss MicroImaging, Germany).

### Laser capture microdissection (LCM), RNA extraction, and RNA sequencing

LCM was performed with a PALM Robot-Microbeam system (Zeiss MicroImaging, Munich, Germany). Cambium microdissected cells were catapulted into the adhesive caps of 500 μl tubes (Supplementary Fig. S2E-K). Total RNA was isolated using the PicoPure RNA Isolation Kit (Thermo Fisher Scientific, Sweden). Quality and quantity of RNA samples were assessed using the Bio-Rad Experion analyser and Experion RNA high-sense analysis kit (Bio-Rad, USA). Total RNA from each biological replicate was amplified using the MessageAmp II aRNA amplification kit (Ambion, Austin, TX, USA). Amplified RNA profiles were verified using the Experion analyser and Experion RNA standard-sense analysis kit (Bio-Rad, USA). In total, 12 cDNA paired-end libraries were generated using the mRNA-Seq assay for transcriptome sequencing on an Illumina HiSeq™ 2000 platform at Beijing Genome Institute (BGI, China), but only 11 were sequenced as one T89 (T_1_) sample failed the quality check.

### Pre-processing of RNA-seq data

The data pre-processing was performed as described in Delhomme *et al.* (2014). Briefly, the quality of the raw sequence data was assessed using FastQC (http://www.bioinformatics.babraham.ac.uk/projects/fastqc/).

Residual ribosomal RNA (rRNA) contamination was assessed and filtered using SortMeRNA (v2.1; Kopylova *et al.*, 2012; settings --log –paired in --fastx--sam --num_alignments 1) using the rRNA sequences provided with SortMeRNA (rfam-5s-database-id98.fasta, rfam-5.8s-database-id98.fasta, silva-arc-16s-database-id95.fasta, silva-bac-16s-database-id85.fasta, silva-euk-18s-database-id95.fasta, silva-arc-23s-database-id98.fasta, silva-bac-23s-database-id98.fasta and silva-euk-28s-database-id98.fasta). Data were then filtered to remove adapters and trimmed for quality using Trimmomatic (v0.32; Bolger *et al.*, 2014; settings TruSeq3-PE-2.fa:2:30:10 LEADING:3 SLIDINGWINDOW:5:20 MINLEN:50). After both filtering steps, FastQC was run again to ensure that no technical artefacts were introduced. Filtered reads were aligned to v3.0 of the *P. trichocarpa* genome (Phytozome) using STAR (v2.5.2b; Dobin *et al.*, 2013; non default settings: --outSAMstrandField intronMotif--readFilesCommand zcat--outSAMmapqUnique 254 --quantMode TranscriptomeSAM --outFilterMultimapNmax 100 --outReadsUnmapped Fastx --chimSegmentMin1--outSAMtype BAM SortedByCoordinate --outWigType bedGraph --alignIntronMax 11000). The annotations obtained from the *P. trichocarpa* v3.0 GFF file were flattened to generate ‘synthetic’ gene models. This synthetic transcript GFF file and the STAR read alignments were used as input to the HTSeq (Anders *et al.*, 2015) htseq-count python utility to calculate exon-based read count values. The htseq-count utility takes only uniquely mapping reads into account.

### Differential gene expression analysis

Statistical analysis of single-gene differential expression between conditions was performed in R (v3.4.0; Team, 2018) using the Bioconductor (v3.5; Gentleman *et al.*, 2004) DESeq2 package (v1.16.1; Love *et al.*, 2014). FDR adjusted *P* values were used to assess significance; a common threshold of 1% was used throughout. For the data quality assessment and visualization, the read counts were normalized using a variance stabilising transformation (vst) as implemented in DESeq2. The biological relevance of the data, such as similarity of biological replicates (Supplementary Fig. S3A,B) and other visualizations (e.g. heat maps), were obtained using custom R scripts, available at https://github.com/nicolasDelhomme/poplarcambium.

The gene list encoding *P. trichocarpa* transcription factors was downloaded from the plant transcription factor database v4.0 (http://planttfdb.gao-lab.org/).

Dendrograms and heat maps were generated using the function heatmap.2 from the gplots R library. Heat maps of differentially expressed genes (DEGs, DE cut-offs of FDR ≤0.01 and |LFC| ≥0.5), were generated using the function heatmap.2 from the gplots R library. The 17 997 genes, which were detected in all biological replicates, were used for further analysis. Genes which were expressed only in one or two biological replicates for each genotype, but which were significant for differential expression between T89 and OP42, were analysed separately. The gene expression mean values are listed in Supplementary Dataset S3 (sheet 6).

### Gene Ontology analysis

The REVIGO web server (http://revigo.irb.hr/) was used to summarize Gene Ontology (GO) terms from differentially expressed genes (Supek *et al.*, 2011). The GO terms with a false discovery rate (FDR; e-value corrected for list size) of ≤0.05 were submitted to the REVIGO tool, and the ‘small allowed similarity’ setting was selected to obtain a compact output of enriched GO terms. The overall significance of enriched processes was expressed as the sum of 100 × –log_10_ (FDR) for each enriched GO term counted within that process. This technique was adapted from the method used to visualise enriched GO terms as a percentage of the total enriched terms in the TreeMap function of the REVIGO web server.

### Identification of poplar homologues of Arabidopsis *ARFs* and *MYC* transcription factors

To identify poplar homologues of Arabidopsis ARFs, the complete amino acid sequences from Arabidopsis AtARF6 (AT1G30330), AtARF8 (AT5G37020) and AtARF17 (AT1G77850), were used in BLAST searches of the *Populus trichocarpa* proteome (https://phytozome.jgi.doe.gov/pz/portal.html) and popgenie (https://popgenie.org/). Full-length amino acid sequences of the selected poplar and Arabidopsis ARFs were subjected to phylogenetic analysis using MEGA8.0 software. The phylogenetic analysis was performed with the MEGA8.0 software using the Neighbor-Joining method on the p-distance model with 1000 iterations. The most closely related orthologues were chosen for the study (Supplementary Fig. S4A). We used poplar *ARF* gene names according to the nomenclature in PopGenIE. Corresponding gene names are as follows: *PtrARF6.1*; Potri.005G207700, *PtrARF6.2*; Potri.002G055000, *PtrARF6.3*; Potri.001G358500, *PtrARF6.4*; Potri.011G091900, *PtrARF8.1*; Potri.004G078200, *PtrARF8.2*; Potri.017G141000, *PtrARF17.1*; Potri.005G171300 and *PtrARF17.2*; Potri.002G089900. Similarly, the poplar homologues of Arabidopsis *AtMYC2.1* were analysed; their corresponding gene names are as follows: *PtrMYC2.1*; Potri.003G092200, *PtrMYC2.2;* Potri.001G142200, *PtrMYC2.3;* Potri.002G176900, *PtrMYC2.4;* Potri.001G083500, *PtrMYC2.5*; Potri.003G147300 and *PtrMYC2.6*; Potri.014G103700.

### Generation of transgenic hybrid aspen plants

To amplify the candidate genes, cDNA was synthesized (SuperScript II Reverse Transcriptase, Thermo Fisher Scientific, USA) starting from total RNA extracted from hybrid aspen T89 (*P. tremula* × *P. tremoloides*) leaves using Spectrum™ Plant Total RNA Kit (Sigma-Aldrich, USA) followed by DNAse treatment (TURBO DNA-free Kit, Ambion). As it is not possible to distinguish the *P. tremula* sequence from that of *P. tremuloides*, the genes are referred to as *PttARF6.4, PttARF8.2, PttARF17.2,* and *PttMYC2.1,* and the corresponding primers used for amplification of the coding sequences are listed in Supplementary Table S1.

The amplified cDNAs of *PttARF6.4, PttARF8.2,* and *PttMYC2.1* were cloned independently into the pENTR/D-TOPO donor vector (Thermo Fisher Scientific, USA) and transferred into the pK2GW7 plant transformation vector (Gateway Vectors, VIB-UGent Center for Plant Systems Biology, Belgium). *PttARF6.4* and *PttARF8.2* coding sequences were also cloned into the pK2GWFS7 vector (Gateway Vectors, VIB-UGent Center for Plant Systems Biology, Belgium) in which the CaMV35S promoter had been replaced by a 2 kb promoter fragment from the *PttHB3a* gene for specific expression in the cambium (Schrader *et al.*, 2004). To down-regulate the *ARF*s genes, we generated RNAi constructs with 578 bp, 624 bp, and 480 bp fragments from *PttARF6.4*, *PttARF8.2*, and *PttARF17.2*, respectively. These fragments were amplified using primers listed in Supplementary Table S1 and T89 cDNA as a template. Due to high coding nucleotide sequence similarity, RNAi constructs targeting both *Ptt*A*RF6.3* and *PttARF6.4* paralogues, *PttARF8.1* and *PttARF8.2* paralogues, or *PttARF17.1* and *PttARF17.2* paralogues were generated. The amplified fragments were cloned into pENTR/D-TOPO (Thermo Fisher Scientific, USA) and then transferred into the plant transformation vector pK7GWIWG2.

All the different constructs were transformed independently into *Agrobacterium tumefaciens* GV3101 pmp90RK, which was used to transform the hybrid aspen T89. In total, 14 independently transformed lines for each construct were generated. The relative expression of *PttARF6.1/2*, *PttARF6.3/4, Ptt*ARF*17.1/2,* and *PttARF17.1/2* in the respective transgenic lines were further quantified by qPCR. Two independent RNAi lines for each construct were selected and analysed for their adventitious rooting ability.

### Quantitative real-time PCR analysis

To determine overexpression or down-regulation of the selected genes in the transgenic lines, five 5 mm stem pieces were taken at the base of cuttings from T89 (three biological replicates) and transgenic lines (three biological replicates for each line) at the time of adventitious rooting assay, and pooled. Each biological replicate was formed by a pool of stem pieces collected from three different plants. Total RNA was extracted using the Spectrum™ Plant Total RNA Kit (Sigma-Aldrich). A total 10 μg of RNA samples was treated with TURBO DNA-free Kit (Ambion) to remove contaminating DNA from RNA preparations, and to remove the DNAse from the samples. cDNA was synthesized using SuperScript® III Reverse Transcriptase Kit (Invitrogen) following the DNase treatment. Quantitative real-time PCR analyses were carried out with a Roche LightCycler 480 II instrument, and expression values were calculated relative to the reference gene expression values, by using the ∆-ct-method, as described previously (Gutierrez *et al.*, 2008). *PtUBIQUITIN* (Potri.001G418500), which had been previously validated for gene expression analysis in T89 stem cuttings with geNORM (Gutierrez *et al.*, 2008) was used as the reference gene. Due to the high sequence similarity we failed to design paralogue-specific qPCR primers, and instead designed primers that specifically amplify *PttARF6.1* and *PttARF6.2* paralogues together (*PttARF6.1/2*), as well as *PttARF6.3* and *PttARF6.4* paralogues together (*PttARF6.3/4*). Similarly, primers were designed for *PttARF8.1* and *PttARF8.2* (*PttARF8.1/2*), and *PttARF17.1* and *PttARF17.2* (*PttARF17.1/2*) paralogue genes. Primers were designed to span the microRNA cleaving site for each gene to quantify the un-cleaved transcripts only (Supplementary Table S1).

### Statistical analysis

Statistical analysis was performed using the GraphPad Prism version 9.0 for Mac (www.graphpad.com). Unless specified one-way ANOVA followed by Tukey’s multiple comparison post-hoc were used to compare means.

## Results

### Hybrid aspen and hybrid poplar show different patterns of adventitious root formation

**Fig. 1.**
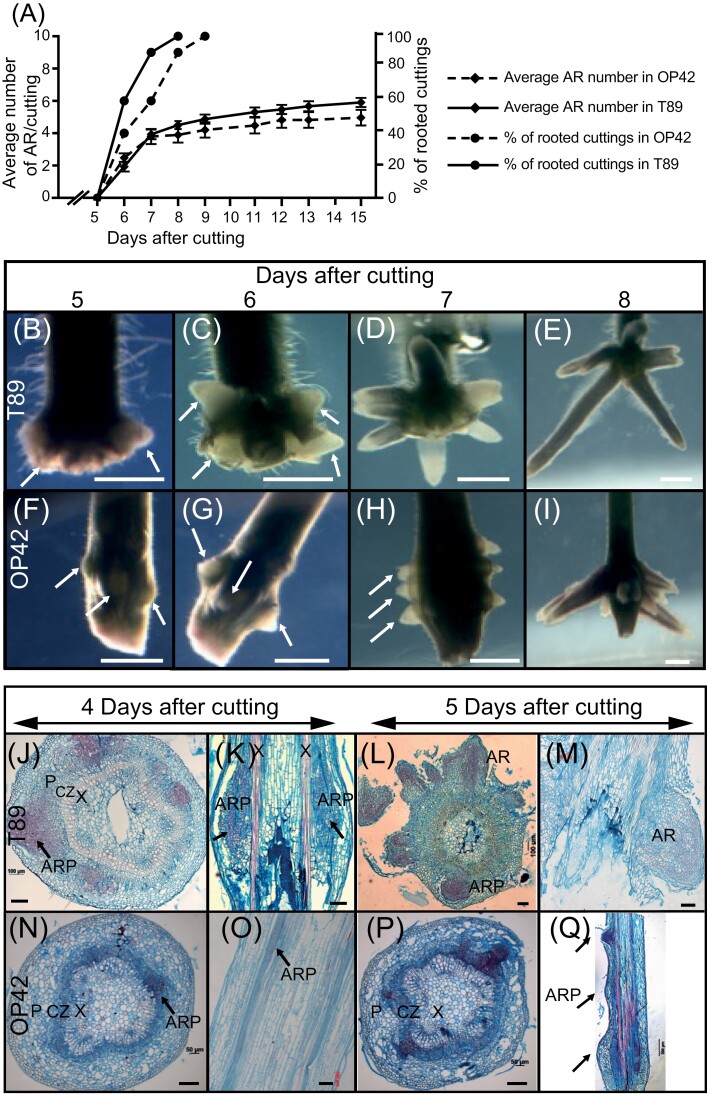
Pattern of adventitious rooting in hybrid aspen and hybrid poplar *in vitro*. (A) Average number of adventitious roots (ARs) and percentage of rooted cuttings in T89 and OP42. Fifteen 3 cm long cuttings, starting from the shoot apex, were taken from 4-week-old plantlets, propagated *in vitro*, and transferred onto half-strength MS medium as shown in Supplementary Fig. S1A, B, D). The emerged ARs were scored starting on day 5 after transfer to fresh medium, until day 15. Data from three independent biological replicates, each of 15 stem cuttings, were pooled and averaged. Error bars indicate standard error. (B to E) Pictures of the base of T89 cuttings taken at day 5, 6, 7 and 8 showing AR emerging primordia (arrows in B, C) and elongating AR (D,E). (F to I) Pictures of the base of OP42 cuttings taken at day 5, 6, 7 and 8 showing AR emerging primordia (arrows in F, G, H) and elongating AR (I). Scale bars in B to I = 2 mm. (J to Q) Cross- (J, L, N, P) and longitudinal (K, M, O, Q) sections show that in both cases the AR primordia develop from cells situated in the cambium/phloem region. Scale bars in J to P = 100 μm CZ = cambial zone; P = Phloem; X = Xylem; ARP = Adventitious root primordium; AR = Adventitious root.

**Fig. 2.**
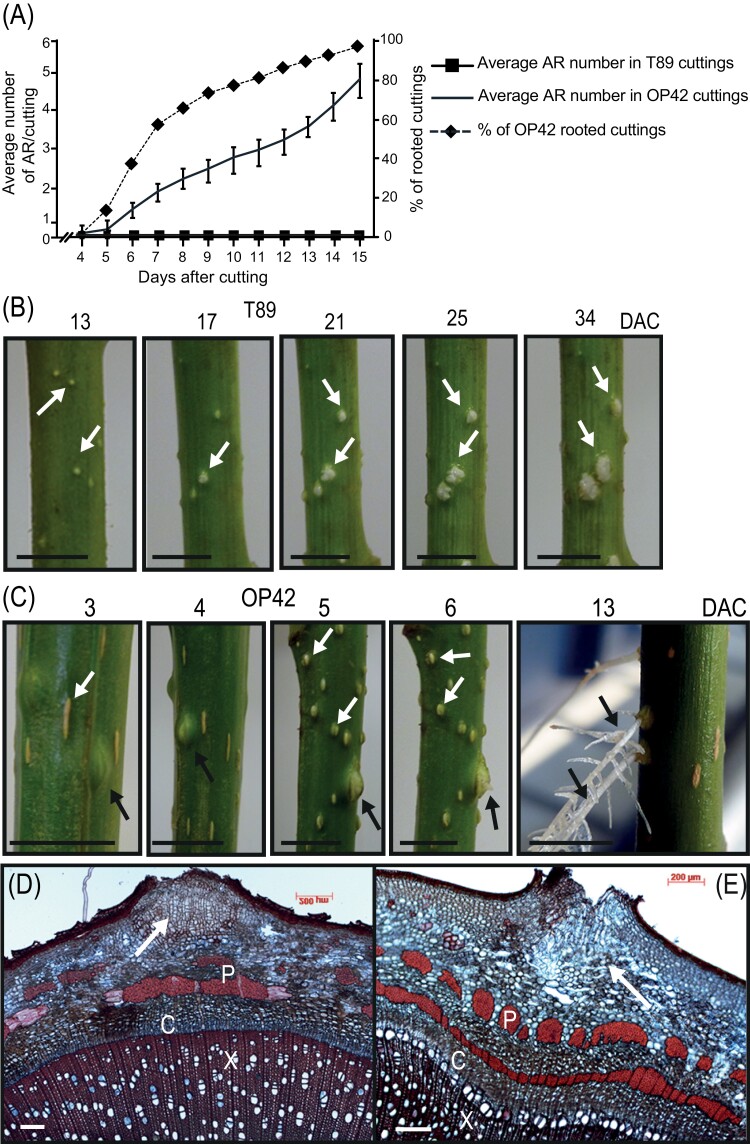
Adventitious root development in woody stem cuttings under hydroponic conditions. (A) Average number of adventitious roots (AR) and rooting percentage in T89 and OP42. About 20 cm lengths of stem from 3-month-old greenhouse-grown hybrid aspen T89 and OP42 plants were used. The stem cuttings were kept in hydroponic conditions for 5 weeks and the number of ARs was scored every day after cutting (DAC). Data from three biological replicates, each of at least 15 stem cuttings, were pooled and averaged. Error bars indicate standard error. (B) In T89 only lenticels were observed (white arrows). (C) In OP42, bulges of AR primordia were observed 3 DAC, and fully developed into ARs at 13 DAC (black arrows). Lenticels were also observed in OP42 cuttings (white arrows). (D, E) Cross-sections at the level of a lenticel (white arrows) in T89 (D) and OP42 (E). X = xylem; C = cambium; P = phloem. Scale bars are 1.5 cm in B and C panels and 200 µm in D and E.

To understand why some genotypes readily develop AR and others do not, we compared the rooting efficiency of cuttings from the poplar clone OP42 (*P. trichocarpa × P. maximowiczii*) and the hybrid aspen clone T89 (*P. tremula × P. tremuloides*) from juvenile plants kept *in vitro* (Fig. 1; Supplementary Fig. S1A, B, D) and from stem cuttings of 3-month-old plants grown in the greenhouse (Fig. 2; Supplementary Fig. S1C, E). When cuttings were taken from juvenile *in vitro* plants, no significant difference was observed between the two clones (Fig. 1A; *P*<0.05). Nevertheless, in T89 *in vitro* cuttings, AR developed at the base of the cuttings in a crown-like arrangement (Fig. 1B-E), while in OP42, AR developed a few mm above the base of the cuttings and along the stem (Fig. 1F-I, O, Q). Cross- and longitudinal sections showed that in both cases, the AR primordia initiated from the cambium region (Fig. 1J-Q) as shown previously in cuttings of the *P. trichocarpa* clone 101-74 (Rigal *et al.*, 2012). In contrast, when cuttings were taken from greenhouse-grown 3-month-old plants (Supplementary Fig. S1C) and kept in a hydroponic culture system as described elsewhere (Merret *et al.*, 2010; Rigal *et al.*, 2012; Supplementary Fig. S1E), T89 cuttings were unable to develop ARs (Fig. 2A, B), while 100% of OP42 cuttings did root (Fig. 2A, C). For OP42 cuttings, the first indication that AR primordia were emerging was the presence of bulges on the stems that were visible as early as 3 d after cutting, and AR emerged after around 5 or 6 d (Fig. 2C), and fully developed and formed secondary roots at 13 d after cutting (Fig. 2C). In the case of T89 we never observed any bulge on the surface of the cuttings, and to check if there were any arrested primordia, cross sections were made at different levels in the stem cuttings 6 and 26 d after being cut. No arrested primordia were observed, suggesting that the repression of AR development occurred at very early stages of AR initiation.

In both T89 and OP42, we observed the formation of lenticels; these correspond to cell proliferation regions in the cortex due to the high humidity in hydroponic conditions (Fig. 2D, E).

### Transcriptomic profile and functional classification of differentially expressed genes from cambium tissue between OP42 and T89 poplar genotypes

To explain the extreme difference in rooting performance, we performed a transcriptomic analysis of the cambium of OP42 and T89 cuttings from 3-month-old plants grown in the greenhouse (Supplementary Fig. S2A). According to Ramirez-Carvajal *et al.* (2009) the highest number of DEGs in stem cuttings of *Populus tremula × Populus alba* was observed between 6 and 24 h after cutting. Therefore, to target the early events of AR initiation, before the occurrence of primordia, we decided to analyse the cambium transcriptome of OP42 and T89 cuttings 24 h after cutting. We performed LCM (Supplementary Fig. S2D-I) to dissect and collect homogenous and specific cambium tissues from the basal 5 mm of stem cuttings at time T_0_ (immediately after cutting; Supplementary Fig. S2B) and T_1_ (24 h after transfer in hydroponic conditions; Supplementary Fig. S2C).

**Fig. 3.**
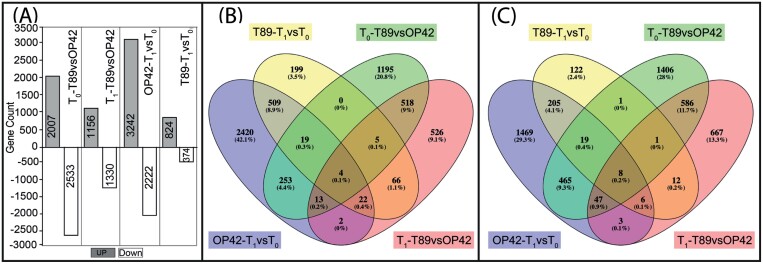
Number of differentially expressed genes (DEGs) between T89 and OP42. (A) Total number of DEGs up- and down-regulated in T89 and OP42. Venn diagram of DEGs between T89 and OP42. (B) Up-regulated genes (C) Down-regulated genes. Abbreviations signify as follows: T_1_-T89-vs-OP42; genes are up- or down-regulated in T89 compared with OP42 at time T_1_; T_0_-T89-vs-OP42; genes are up- or down-regulated in T89 compared with OP42 at time T_0_; T89-T_1_-vs-T_0_; genes are up- or down-regulated at time T_1_ compared with time T_0_ in T89. OP42-T_1_-vs-T_0_; genes are up- or down-regulated at time T_1_ compared with time T_0_ in OP42.

We mapped the RNA-seq reads to the *P. trichocarpa* reference genome (Supplementary Dataset S1, sheet1) and classified 17 997 genes in the current annotation as being expressed significantly in all biological replicates in both genotypes at times T_0_ and T_1_ (Supplementary Dataset S1, sheet 2). These 17 997 genes represent approximately 43% of the annotated genes in the *Populus* genome (poplar v3 assembly version; Tuskan *et al.*, 2006). Interestingly, there were more DEGs in OP42 after 24 h in hydroponic conditions than in T89 (Fig. 3). In the case of T89, a total of 1198 (6.6% of the 17 997) genes were differentially expressed; 824 were up-regulated and 374 were down-regulated at T_1_ compared with T_0_ (Fig. 3A; Supplementary Dataset S2, sheets 11–13). GO enrichment analysis of DEGs showed a significant enrichment of GO terms related to biological processes, and molecular functions related to carbohydrate catabolism or redox mechanisms, regulation of transcription, response to abiotic stresses, cation binding, nucleic acid binding activity, or electron carrier activity (Supplementary Dataset S3, sheets 4, 5). In contrast, in OP42, a total of 5464 genes (30% of the 17 997 genes) were found to be differentially expressed, among which 3242 were up-regulated, and 2222 down-regulated at time T_1_ compared with T_0_ (Fig. 3A, C; Supplementary Dataset S2, sheets 8–10). Interestingly, among the 3242 DEGs, 2420 (74.6%) were exclusively up-regulated in OP42 at T_1_ (Fig. 3B), suggesting a specific remodulation of the transcriptome in OP42 during the 24 h timeframe that did not occur in T89. The GO enrichment analysis of these up-regulated DEGs showed a significant enrichment of GO in cellular components, biological processes or molecular functions related to cell metabolism or cell biology, such as transcription regulation, translation and post translation regulation (Supplementary Dataset S3, sheet 4). Similarly, 66% of the 2222 DEGs that were down-regulated in OP42 at T_1_ compared with T_0_ were specifically differentially expressed in OP42 (Fig. 3C). In contrast to the up-regulated genes, the GO enrichment analysis showed a significant enrichment of GO in cellular components, biological processes or molecular functions related to abiotic stress responses (Supplementary Dataset S3, sheet 5). When the two genotypes were compared with each other, 25% of the 17 997 genes were differentially expressed between OP42 and T89 at T_0_ (Fig. 3A; Supplementary Dataset S2) among which, 2007 were up-regulated in T89 compared with OP42 (Fig. 3A) while 2533 were down-regulated (Fig. 3A; Supplementary Dataset S2, sheets 2 to 4). This difference between the two genotypes was reduced to 14% 24 h after transfer into hydroponic conditions, with 1156 up-regulated and 1330 down-regulated in T89 compared with OP42 (Fig. 3A; Supplementary Dataset S2, sheets 5 to 7). The genes that were differentially expressed between T89 and OP42 are mostly involved in cellular and chemical homeostasis, photosynthesis, dioxygenase activity and protein synthesis (Supplementary Dataset S3, sheets 4 and 5).

### Genes related to cambium or vascular tissues behave similarly in both genotypes

After checking the similarity of the biological replicates of RNA-seq data (Supplementary Fig. S3A, B), we also confirmed the quality and the specificity of the datasets. For this, we selected a list of 40 Arabidopsis genes described as being expressed in the cambium or vascular tissues, and checked the expression of their putative *Populus* orthologues in our data (Supplementary Fig. S3C; Supplementary Dataset S3, sheet 1). All were found to be expressed (and most behaved similarly) in the two genotypes, showing a slight up-regulation or down-regulation in both OP42 and T89 between T_0_ and T_1_ (Supplementary Fig. S3C; Supplementary Dataset S3, sheet 1). A few exceptions to this general pattern included Potri.003G111500 (*PtrPPNRT1.2*), Potri.004G223900 (similar to *AtCLAVATA1-related* gene) and Potri.014G025300 (similar to *AtWOX4b*), which were slightly down-regulated in T89 but up-regulated in OP42 24 h after cutting; additionally, a few genes were up-regulated in T89 compared with OP42 at T_0_ and T_1_. They comprise Potri.003G111500 (*PtrPPNRT1.2*), Potri.001G131800 (similar to Arabidopsis *BREVIS RADIX* gene) and Potri.002G024700 (ARF5), Potri.009G017700, which is similar to *AtLONESOME HIGHWAY*, a bHLH master transcriptional regulator of the initial process of vascular development.

### Genes encoding reactive oxygen species scavenging proteins are mostly up-regulated in OP42 compared with T89

Reactive oxygen species (ROS) are signalling molecules involved in the response to biotic and abiotic stresses as well as many aspects of plant development, including AR formation, as shown by recent studies (reviewed in Nag *et al.*, 2013; Li *et al.*, 2017; Velada *et al.*, 2018). We therefore searched for genes encoding ROS scavenging proteins among all DEGs in T89 and OP42. We identified 43 DEGs encoding ROS scavenging proteins, 33 of which belong to the GLUTATHIONE S-TRANSFERASE superfamily (GSTs) and 10 to the PEROXIDASE superfamily (Supplementary Dataset S3 sheet 3). Twenty of these genes were up-regulated at T_1_ compared with T_0_ in both genotypes, but on average the fold change was higher in OP42 than in T89 (Supplementary Fig. S5; Dataset S3, sheet 3); nine genes were repressed 24 h after cutting in both genotypes. The most striking observation was that 32 out of 43 genes were significantly up-regulated in OP42 compared with T89 at T_1_, and 21 of those were also up-regulated in OP42 at T_0_ (Supplementary Dataset S3, sheet 3); only six were up-regulated in T89 compared with OP42 at T_0_ and T_1_; four were up-regulated in T89 compared with OP42 at T_0_, but down-regulated in T89 compared with OP42 at T_1_; and five were up-regulated in OP42 compared with T89 at T_0_ - but by contrast, up-regulated in T89 at T_1_.

### The easy-to-root OP42 shows increased transcriptional activity in the cambium compared with the difficult-to-root T89

The different stages of AR initiation (ARI) in *Populus* are associated with substantial remodelling of the transcriptome (Ramirez-Carvajal *et al.*, 2009; Rigal *et al.*, 2012). We therefore focused our analysis on the expression of TFs. From the 58 families of TFs identified in *Populus*, 49 families were represented in the DEG list (Table 1; Supplementary Dataset S2, Dataset S3, sheet 2) and most of the DEGs were observed in OP42 (Table 1). Furthermore, 24 h after cutting, 210 and 209 TFs were up- or down-regulated respectively in OP42, while in T89 there were only 89 up-regulated and 43 down-regulated DEGs (Table 1). The most represented DEGs belong to the *ARF*, *bHLH*, bZIP, *C2H2-* and *C3H-* type zinc-finger family, *ERF*, *LBD*, *MYB*, *MYB-related*, *NAC* and *WRKY* families. Several genes belonging to these TF families have been shown to be involved in the control of adventitious rooting in *Populus* species (reviewed in Legue *et al.*, 2014).

**Table 1.** Numbers of differentially expressed transcription factors which were either up- or down-regulated in T89 and OP42.

Families	T89 T1-vs-T0		OP42 T1-vs-T0		T0 T89-vs-OP42		T0 T89-vs-OP42	
	Up-regulated	Down-regulated	Up-regulated	Down-regulated	Up-regulated	Down-regulated	Up-regulated	Down-regulated
AP2	0	1	2	2	2	1	2	1
ARF	2	3	0	12	5	5	1	0
ARR-B	0	0	0	1	1	0	0	0
B3	0	1	1	4	8	3	6	2
BBR-BPC	0	0	0	1	0	2	0	0
BES1	0	0	3	0	0	0	1	0
bHLH	15	2	32	14	3	14	7	6
bZIP	3	1	6	11	7	8	6	5
C2H2	4	2	10	6	7	9	0	6
C3H	0	2	3	9	5	2	1	2
CAMTA	0	0	0	1	0	1	1	0
CO-like	0	1	0	3	2	0	3	0
CPP	0	0	0	0	0	1	0	0
DBB	0	1	1	4	0	1	0	0
Dof	1	3	3	7	1	3	1	0
EIL	1	0	0	0	1	0	1	0
ERF	21	5	42	12	7	14	4	7
FAR1	0	0	0	0	1	0	0	1
G2-like	1	0	2	7	3	5	1	0
GATA	1	1	4	2	3	3	0	1
GeBP	0	0	1	0	0	0	0	0
GRAS	1	1	3	8	3	2	3	1
GRF	0	1	1	5	0	3	0	0
HB-other	0	0	0	2	0	1	0	0
HD-ZIP	2	0	6	6	3	0	3	1
HRT-like	0	0	0	1	0	0	0	0
HSF	1	1	5	2	4	3	1	1
LBD	3	0	11	2	1	3	3	2
LSD	0	0	1	0	0	0	1	1
M-type_MADS	0	0	1	1	0	3	0	1
MIKC_MADS	2	3	0	5	4	7	4	1
MYB	7	2	19	13	4	10	2	1
MYB_related	1	4	5	16	10	7	4	3
NAC	3	1	8	16	6	8	4	1
NF-YA	0	3	0	9	2	0	1	0
NF-YB	0	1	0	2	2	2	1	1
NF-YC	0	0	1	0	0	1	0	0
Nin-like	0	0	0	1	2	0	1	0
SBP	0	3	1	7	2	3	0	0
TALE	0	0	1	7	1	1	1	1
RAV	1	0	2	0	0	0	0	0
S1Fa-like	0	0	1	0	0	1	0	0
TCP	1	0	0	3	2	1	0	0
Trihelix	5	0	7	2	5	1	4	0
WOX	0	0	2	0	1	0	0	0
VOZ	0	0	0	0	0	1	0	0
WRKY	13	0	23	3	4	6	3	2
ZF-HD	0	0	2	0	3	1	0	1
YABBY	0	0	0	2	0	0	0	0
**Total**	**89**	**43**	**210**	**209**	**115**	**137**	**71**	**49**

Genes from the *LATERAL BOUNDARY (LBD)* gene family have been shown to be involved in the development of lateral organs in Arabidopsis (reviewed in Matsumura *et al.*, 2009). In particular *AtLBD16*, *AtLBD17, AtLBD18* and *AtLBD29* were shown to be involved in lateral root, adventitious root or regeneration processes in Arabidopsis (Okushima *et al.*, 2007; Liu *et al.*, 2018). Interestingly we observed that 10 *PtrLBD* genes were specifically up-regulated at T_1_ in OP42, among which the putative orthologue of *AtLBD16*, *PtrLBD16* (Potri. 002G041200), was up-regulated in OP42 at T_1_ with a log_2_ FC of 4.3 (Supplementary Dataset S2, sheet 6). In addition, *PtrLBD11* (Potri.010G217700) was also up-regulated in OP42 at T_1_ with a log_2_ FC of 8.5 (Supplementary Dataset S2, sheet 6). *PtrLBD11* is the putative orthologue of *AtLBD11* which was shown to be involved in secondary growth and stem cell maintenance in the cambium during root development (Ye *et al.*, 2021). The expression of other genes involved in the control of vascular differentiation that could contribute to the rooting difference between T89 and OP42 were specifically up- or down-regulated in OP42.

The NAC family of transcription factors is one of the largest plant-specific families of transcriptional regulators involved in various aspects of plant development and responses to biotic and abiotic stresses (reviewed in Olsen *et al.*, 2005). Twenty-four genes from the *NAC* family were differentially expressed in OP42 at T_1_ compared with T_0_ (Table 1; Supplementary Dataset S2, sheet 6). Among the up-regulated genes encoding NAC transcription factors in OP42, Potri.001G080900 (log_2_ FC of 7.5) and Potri.002G057200 (log_2_ FC of 9) encode putative orthologues of *AtJUNGBRUNNEN1* (*AtJUB1/AtNAC042*), a transcription factor induced by ROS, and that represses senescence in Arabidopsis (Wu *et al*, 2012). This up-regulation could be related to the up-regulation of genes encoding ROS scavenging proteins, as described above. Fifteen *NAC* genes were specifically down-regulated in OP42 (Supplementary Dataset S2, sheet 6). Potri.001G404400 and Potri.017G063300 were down-regulated with a log_2_ FC of –3 and –2, respectively. These two genes encode putative orthologues of the Arabidopsis VND-INTERACTING2 (AtVNI2/AtNAC83) protein which was shown to interact with the AtVASCULAR-RELATED NAC-DOMAIN7 (AtVND7) transcription factor regulating the differentiation of xylem vessels (Yamaguchi *et al.*, 2008) and to repress its activity (Yamaguchi *et al.*, 2010). A third putative orthologue of *AtVNI2* (Potri.003G166500) was in contrast up-regulated with a log_2_ FC of 2.6 (Supplementary Dataset S2, sheet 6). Potri.001G404400 and Potri.011G121300 encode two other NAC transcription factors involved in vascular development, both of which were also down-regulated in OP42 at T_1_ (Supplementary Dataset S2, sheet 6). Potri.001G404400 is a putative ortholog of *AtNAC-REGULATED SEED MORPHOLOGY 1 (AtNARS1/AtNAC2*) which was shown to be involved in the regulation of asymmetric cell divisions of sieve element precursors in the phloem downstream of *AtSHORTROOT* (*AtSHR*), a GRAS family TF (Kim *et al.*, 2020). Potri.011G121300 encodes a putative orthologue of *AtNAC86* involved in the differentiation of sieve elements (Furuta *et al.*, 2014). Interestingly Potri.007G132000, the orthologue of *AtSHR*, was up-regulated in OP42 at T_1_ with log_2_ FC of 3 (Supplementary Dataset S2, sheet 6). In addition, seven *SCARECROW-Like* (*SCL*) genes of unknown function were down-regulated in OP42 at T_1_ (Supplementary Dataset S2, sheet 6). In Arabidopsis, *AtSHR* together with its closely related member *AtSCARECROW* (*AtSCR*) controls radial patterning during root development (Nakajima *et al.*, 2001). They are also important for the maintenance of the root apical meristem and the quiescent centre (reviewed in Vernoux and Benfey, 2005) as well as the positioning of the stem cell niche (Lucas *et al.*, 2011). In *Pinus radiata* the expression of several *PrSCL* genes was associated with the maturation-related decline of competence to develop adventitious roots (Abarca *et al.*, 2014). In addition, several *SCARECROW-LIKE* (*SCL*) family genes, such as *PrSCL1* from *Pinus radiata* and *CsSCL1* from *Castanea sativa* (Sanchez *et al.*, 2007; Solé *et al.*, 2008; Vielba *et al.*, 2011), *PrSHR* from *Populus radiata* (Solé *et al.*, 2008), and *PtrSCR* from *Populus trichocarpa* (Rigal *et al.*, 2012), were shown to be induced during the earliest stages of AR formation in cuttings generated *in vitro*. In OP42, Potri.001G242000, which is similar to *AtSCL30/SCL14,* an essential gene for the activation of stress induced response (Fode *et al.* 2008), was up-regulated with log_2_ FC of 9 (Supplementary Dataset S2, sheet 6). In T89, three *SCL* genes encoding DELLA proteins involved in the gibberellic acid signalling pathway were up-regulated compared with OP42 at T_1_ (Supplementary Dataset S2, sheet 6). Gibberellic acid has been shown to be a negative regulator of adventitious root development in *Populus* (Mauriat *et al.*, 2014). Whether a difference in the regulation of gibberellic acid signalling pathway explains the rooting difference between OP42 and T89, requires further investigation.

The *APETALA2/ETHYLENE RESPONSE FACTOR* (*AP2/ERF*) family was the most represented, with 21 and 42 *ERF* genes up-regulated at T_1_ in T89 and OP42, respectively (Table 1; Supplementary Dataset S3, sheet 2). Twenty of the *ERF*s up-regulated in T89 were also up-regulated in OP42 at 24 h after cutting. Among the 22 genes specifically up-regulated in OP42, we found *PtrERF003* (Potri.018G085700; log_2_ FC=7.7; Supplementary Dataset S3, sheet 2) which has been shown to be a positive regulator of AR development in *Populus* (Trupiano *et al.*, 2013), and *PtrERF39* (Potri.003G071700), a likely orthologue of the oxygen sensing *AtRAP2.12* (At1g53910) which has recently been shown to be involved in primary root growth inhibition upon oxygen deficiency in Arabidopsis (Shukla *et al.*, 2020).

Several *WUSHEL-Like Homeobox* genes, have been shown to positively control AR development in *Populus* species (Li *et al.*, 2018; B. Liu *et al.*, 2014; J. Liu *et al.*, 2014; Xu *et al.*, 2015). More specifically, the *P. tomentosa PtoWOX5a* (Potri.008G065400) gene (Li *et al.*, 2018), and the *Populus* × *euramericana PeWOX11/12ba* (Potri.013G066900) and *PeWOX11/12b* (Potri.019G040800) genes (Xu *et al.*, 2015) are involved in AR development in poplar; nevertheless, they were not expressed in the cambium cells of OP42 or T89 (Supplementary Dataset S1). In contrast, we found that two paralogues of *PtrWOX13*, *PtrWOX13a* (Potri.005G101800) and *PtrWOX13b* (Potri.005G252800), were up-regulated in OP42 at 24 h after cutting and transfer into hydroponic conditions (Supplementary Dataset S3, sheet 2). *PtrWOX13* belongs to an ancient clade of *PtrWOX* genes (B. Liu *et al.*, 2014b) and the Arabidopsis *AtWOX13* and *AtWOX14* are involved in the regulation of primary and lateral root development in Arabidopsis (Deveaux *et al.*, 2008).

Recently Wei *et al.* (2020) showed that the *P. ussuriensis PuHox52* gene, which belongs to the HD-Zip sub-family of TFs, positively controls adventitious rooting in *P. ussuriensis*. It acts by inducing nine regulatory hubs, including the jasmonic acid (JA) signalling pathway *PuMYC2* gene (MH644082; Potri.002G176900), a TF from the *bHLH* family, which has been demonstrated to be a positive regulator of AR development in *P. ussuriensis*. In contrast, in our dataset, we found that *P. trichocarpa PtrHox52* (Potri.014G103000) was down-regulated in the cambium of the easy-to-root genotype OP42 at T_1_, i.e. 24 h after cutting and transfer to hydroponic conditions (Supplementary Dataset 3, sheet 2). *PtrHox52* was also up-regulated in the difficult-to-root genotype T89 compared with OP42 at T_1_ (Supplementary Dataset S3, sheet 2). Accordingly, we observed that *PtrMYC2.5* (Potri.003G147300) was up-regulated in the cambium of T89 compared with OP42 at T_1_. There are six paralogues of *MYC2* in *Populus*. Three of these paralogues - *PtrMYC2.1* (Potri.003G092200), *PtrMYC2.2* (Potri.001G142200), and *PtrMYC2.4* (Potri.001G083500), were up-regulated in both T89 and OP42 at T_1_, but with a higher fold change in T89, while *PtrMYC2.5* (Potri.003G147300) was exclusively up-regulated in T89 at T_1_, which led to a significant increase in *PtrMYC2* expression in T89 compared with OP42 (Supplementary Dataset S3, sheet 2). The potential up-regulation of JA signalling in T89 was corroborated by a higher fold change in the expression of several JA-inducible *JA ZIM DOMAIN* (JAZ) genes 24 h after cutting in T89, compared with OP42. *PtrJAZ6* (Potri.003G068900), *PtrJAZ8* (Potri.011G083900) and *PtrJAZ10* (Potri.001G062500) were up-regulated in T89 compared with OP42 at T_1_, with a respective log_2_ FC of 4.25, 5.5, and 4.7 (Supplementary Dataset S2, sheet 6). These results suggest a negative role of JA signalling on AR development, as described in Arabidopsis (Gutierrez *et al.*, 2012; Lakehal *et al.*, 2020a) and contradict the positive role of JA on AR development, as described for *P. ussuriensis* (Wei *et al.*, 2020).

Several genes from the *AUXIN RESPONSE FACTOR* (*ARF*) family have been shown to be involved in AR development in Arabidopsis and *Populus* (Gutierrez *et al.*, 2009, 2012; Cai *et al.*, 2019; Lakehal *et al.*, 2019; Liu *et al.*, 2020). AtARF6 and AtARF8 are positive regulators of adventitious root initiation (ARI), while AtARF17 negatively regulates adventitious rooting (Gutierrez *et al.*, 2009). In *Populus*, PeARF8 also positively regulates AR formation (Cai *et al.*, 2019) but PeARF17, in contrast to the Arabidopsis gene, acts as a positive regulator of AR development in the hybrid poplar *P. davidiana × P. bolleana* (Liu *et al.*, 2020). We identified 36 *PtrARF* genes encoding paralogues of 15 out of the 27 Arabidopsis *ARF* orthologues. Although some of them were more significantly down-regulated in OP42 than in T89 24 h after cutting, they mostly behaved in a similar manner in both genotypes (Supplementary Fig. S6; Supplementary Dataset S3, sheet 6). In particular, expression of *PtrARF6.2* (Potri.002G055000) and *PtrARF6.3* (Potri.001G358500) was up-regulated, while *PtrARF6.1*(Potri.005G207700) and *PtrARF6.4* (Potri.011G091900) were down-regulated in both T89 and OP42 at T_1_ compared with T_0_ (Supplementary Fig. S6; Supplementary Dataset S3, sheet 6). Similarly, both *PtrARF8.1* (Potri.004G078200) and *PtrARF8.2* (Potri.017G141000) were down-regulated at time T_1_ compared with T_0_ in both T89 and OP42. Interestingly, *PtrARF17.1* (Potri.002G089900) was significantly less expressed in the cambium of the difficult-to-root T89 than in OP42, at both T_0_ and T_1_, which agrees with a potential positive role of *PtARF17.1* in AR development.

### *PttARF6* and *PttARF8* positively control, while *PttARF17* negatively controls, adventitious rooting in hybrid aspen

To assess the role of *PttARF*6, *PttARF*8, and *PttARF17* in adventitious rooting in *Populus*, we produced transgenic plants that either overexpressed these genes or down-regulated their expression. Using the PopGenIE data base (http://popgenie.org) we identified the *Populus* genes most closely related to the corresponding Arabidopsis genes (Supplementary Fig. S4A), and checked their expression pattern in the cambium and wood-forming region in the PopGenie database (http://aspwood.popgenie.org/aspwood-v3.0/; Sundell *et al.*, 2017). AspWood provides high resolution *in silico* transcript expression profiling of the genes expressed over the phloem, cambium, and other xylem development zones in aspen trees. We observed, *PtrARF6.1/2/3/4* and *PtrARF8.1/2* to be highly expressed in the phloem/cambium region, while *PtrRF17.1/2* exhibited very low expression in the same region (Supplementary Fig. S4B-D).

**Fig. 4.**
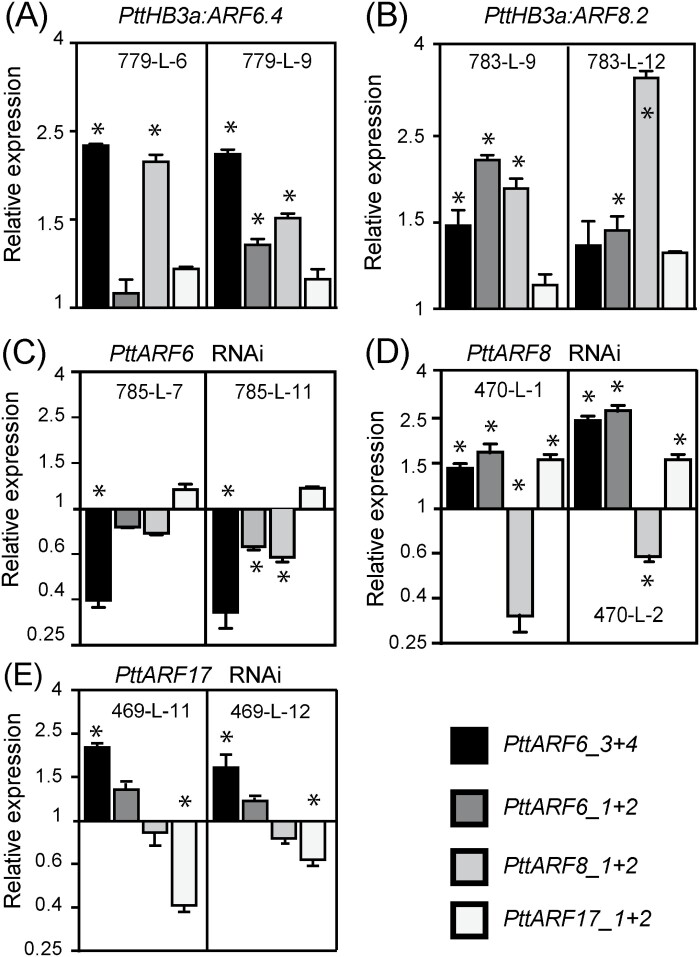
Relative un-cleaved transcript amount of *PtARF6.1/2*, *PtARF6.3/4*, *PtARF8.1/2,* and *PtARF17.1/2* in transgenic lines overexpressing or down-regulated for *PtARF6*, *PtARF8* or *PtARF17.* (A, B) The *PtARF6.1/2*, *PtARF6.3/4*, *PtARF8.1/2*, *PtARF17.1/2* un-cleaved transcript abundance was quantified in stem cutting fragments of independent overexpressing lines (779-L-6, 779-L-9, 783-L-9, 783-L-12) or down-regulated lines (785-L-7, 785-L-11, 470-L-1, 470-L-2, 469-L-11, 469-L-12) (C-E). Gene expression values are relative to the reference gene and calibrated towards the expression in the control line T89, for which the value is set to 1. Error bars indicate SE obtained from three independent biological replicates. A one-way analysis of variance combined with the Dunnett’s comparison post-test indicated that the values marked with an asterisk differed significantly from T89 values (*P*<0.05; *n*=3).

For the lines overexpressing *PttARF6.4* and *PttARF8.1*, coding sequences were cloned under the control of the 35S promoter of the Cauliflower Mosaic Virus (CaMV) or the promotor of the cambium specific gene *PtrHB3a* (Schrader *et al.*, 2004). For down-regulated lines, RNAi constructs were made to target *PttARF6.3* and *4*, *PttARF8.1* and *2*, and *PttARF17.1* and *2* paralogues. We had previously shown that in Arabidopsis hypocotyl, *AtARF6*, *AtARF8* and *AtARF17* regulate the expression of each other at the transcriptional and post-transcriptional level, and that the balance between positive and negative regulators determined the average number of ARs (Gutierrez *et al.*, 2009). As in Arabidopsis, the *Populus ARFs* are regulated by microRNAs (Cai *et al.*, 2019; Liu *et al.*, 2020). We therefore checked the relative transcript amount of the un-cleaved transcript of the three *ARF* types in each transgenic line. A multiple sequence alignment analysis revealed that the coding sequences (CDS) of *PttARF6.1* and *PttARF*6.2 paralogues were highly similar, and we were unable to differentiate their expression by qPCR. A similar situation occurred with *PttARF6.3* and *PttARF6.4*, *PttARF8.1* and *Ptt*A*RF*8.2, *PttARF17.1* and *PttARF17.2*. We therefore designed primers to span the microRNA cleaving site and measured the cumulative expression of the two paralogues (designated *PttARF6_1 + 2*; *PttARF2_3 + 4*; and *PttARF17_1 + 2*) (Fig. 4; Supplementary Fig. S7A, B).

We confirmed the overexpression of *PttARF6_3 + 4* and *PttARF8_1 + 2* in the overexpressing lines (Fig. 4A, B; Supplementary Fig. S7A, B), and the down-regulation of *PttARF6_3 + 4, PttARF8_1 + 2* and *PttARF17_1 + 2* in the RNAi lines (Fig. 4C-E). Interestingly, we observed that, as in Arabidopsis, when the expression of one of the three ARFs was modified, the expression of the others was also affected, establishing a different ratio between potential positive and negative regulators (Fig. 4; Supplementary Fig. S7).

**Fig. 5.**
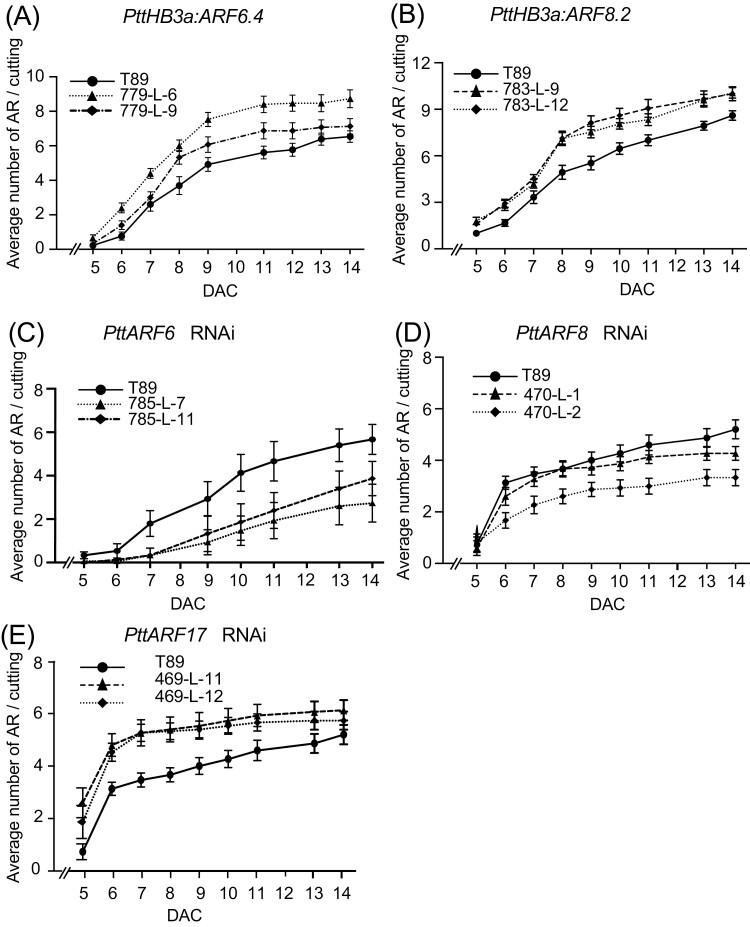
*PtARF6* and *PtARF8* positively control adventitious root (AR) development while *PtARF17* is a negative regulator. (A, B) Average number of ARs on cuttings of transgenic plants expressing *PtARF6.4* (A) and *PtARF8.2* (B) under the cambium specific promoter p*PtHB3.* Rooting assay was performed as described in Material and Methods. Two independent transgenic lines were compared with the control T89. AR number was scored every day starting at day 5 after cutting (DAC) until 14 DAC. For each line, 15 cuttings were analysed. (C-E) Average number of ARs on cuttings of transgenic plants expressing the *p35S:PtARF6.2-RNAi* (C), *p35S*:*PtARF8.4-RNAi* (D) or p35S:*PtARF17.2-RNAi* (E) constructs. Two independent transgenic lines were compared with the T89 control. AR number was scored every day starting at day 5 until 14 DAC. For each line 15 cuttings were analysed. Data are means ±SE, *n*=15, corresponding to two independent lines per construct. A two-way ANOVA followed by Tukey’s multiple comparisons test indicated that the difference between the transgenic lines and the control were significant, except for *PtHB3a:ARF6.4* line 779-L-9 for which the difference was significant only from day 8 to 12, and *PtARF8-RNAi* L-1 for which no significant difference was observed.

We performed rooting assays to assess the ability of the different transgenic lines to produce AR. When either *PttARF6.4* or *PttARF8.2* was overexpressed in the cambium under the control of the *PttHB3* promoter, the transgenic lines produced more AR than the control T89 (Fig. 5A, B). Similar results were obtained with *PttARF6.4* overexpressed under the control of the 35S promotor (Supplementary Fig. S7C), but not with *p35SPttARF8.2* (Supplementary Fig. S7D). The positive effect of *PttARF6* and *PttARF8* was confirmed in the RNAi lines, which produced fewer ARs than the control line T89 (Fig. 5C, D). The role of PttARF17 was unclear, although it has been described as a positive regulator in the hybrid poplar *P. davidiana × P. bolleana* (Liu *et al.*, 2020). However, our results show that when *PttARF17_1 + 2* are down-regulated, the transgenic lines produce more ARs (Fig. 5E), suggesting that PttARF17.1 or PttARF17.2 could be negative regulators. Nevertheless, because *PttARF6_3 + 4* were up-regulated in the *PttARF17* RNAi lines (Fig. 4E), it is difficult to conclude whether the increased AR average number was solely due to the down-regulation of *PttARF17*, the overexpression of *PttARF6_3 + 4,* or to a combination of both.

### PtMYC2.1 is a negative regulator of adventitious root development in hybrid aspen

**Fig. 6.**
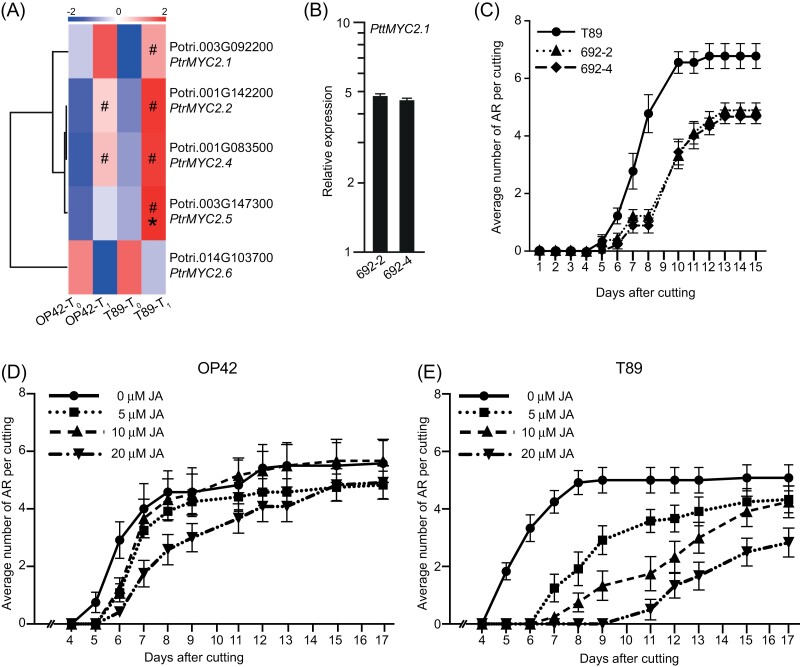
Jasmonate is a negative regulator of AR development in hybrid aspen cuttings. (A) The expression of five out of six *PtMYC2* paralogues found in the transcriptomic dataset presented as a heat map clustering in T89 and OP42 at times T_0_ and T_1_. Colours indicate low expressed genes (blue) or highly expressed genes (red). (B) *PtMYC2.1* transcript abundance was quantified in stem cutting fragments of two independent transgenic T89 lines overexpressing *PtMYC2.1* under the 35S promotor (lines 692-2 and 692-3). Gene expression values are relative to the reference gene and calibrated toward the expression in the control line T89, for which the value is set to 1. Error bars indicate SE obtained from three independent biological replicates. (C) Average number of ARs in stem cuttings of overexpressing *PtMYC2.1* transgenic T89 compared with the wild type T89. For each line 15 cuttings were analysed. Data are means ± SE, *n*=15. (D, E) Average number of ARs in stem cuttings of (D) OP42 and (E) T89 in the absence or presence of 5 µM, 10 µM and 20 µM methyl jasmonate. For each line and each condition, 15 cuttings were analysed. Data are means ±SE, *n*=15. Three independent biological replicates were used. A two-way ANOVA with a Tukey’s multiple comparisons test indicated that in the case of OP42 a significant difference between non-treated plants and treated plants was observed at day 6 for all JA concentrations (*P*<0.05 for 5 µM and 10 μM JA, *P*<0.0001 for 20 μM JA) and then at day 7 and 8 only in the presence of 20 µM JA (*P*<0.01). For T89 a very significant effect of JA was observed for all concentrations from day 5 until day 15 (*P*<0.0001 for 10 µM and 20 μM, *P*<0.05 from day 5 until day 12).

In Arabidopsis, the *AtARF6*, *AtARF8,* and *AtARF17* genes have been shown to act upstream of AtMYC2, which is a negative regulator of AR development (Gutierrez *et al.*, 2012; Lakehal *et al.*, 2020a). In our present study, five out of the six *PtrMYC2* paralogues are shown to be among the DEGs (Fig. 6A; Supplementary Dataset S3, sheet 2). They mostly behaved the same way in both T89 and OP42, but the fold change induction was higher for four of them at T_1_ in the difficult-to-root genotype T89, and *PtMYC2.5* was significantly up-regulated in T89 compared with OP42 at 24 h after cutting (Fig. 6A; Supplementary Dataset S3, sheet 2). These results suggest that PtrMYC2 could be a negative regulator of adventitious rooting in hybrid aspen. To confirm this hypothesis, we produced transgenic hybrid aspen trees overexpressing *PttMYC2.1* under the control of the 35S promoter. The overexpression was confirmed in two independent transgenic lines by qPCR (Fig. 6B), and the rooting assays confirmed that overexpressing *PttMYC2.1* repressed AR development (Fig. 6C). The up-regulation of the JA signalling pathway in T89 cambium compared with OP42 could contribute to the recalcitrance of stem cuttings from greenhouse-grown plants to produce AR. This led us to compare the behaviour of OP42 and T89 in response to exogenous JA. Rooting assays were performed with *in vitro* propagated T89 and OP42 plants in the absence or presence of increasing concentrations of JA (Fig. 6C, D). We observed that even though the two genotypes rooted similarly and responded similarly to exogenous auxin (Supplementary Fig. S8) under *in vitro* conditions, they showed a different response to exogenous JA. The difficult-to-root T89 was more sensitive to exogenously applied JA compared with OP42 (Fig. 6D, E).

## Discussion

*Populus* species are among the most economically utilized trees. Their ability to be propagated vegetatively means that novel genotypes can be rapidly multiplied. Nevertheless, tree cloning is often limited by the difficulty of developing ARs from stem cuttings. Adventitious rooting is a complex multifactorial process. Many QTLs have been detected for adventitious rooting-related traits (Ribeiro *et al.*, 2016; Sun *et al.*, 2019; Zhang *et al.*, 2009), highlighting the genetic complexity of this trait. With the emergence of Arabidopsis as a genetic model, many genes and signalling pathways involved in the control of AR development have been identified (Sorin *et al.*, 2005; Gutierrez *et al.*, 2009, 2012; B. Liu *et al.*, 2014; Hu and Xu, 2016; Lakehal *et al.*, 2019, 2020a, b), and lately, several groups have focused on AR development in *Populus* and identified genes and gene networks involved in this process (Ramirez-Carvajal *et al.*, 2009; Trupiano *et al.*, 2013; Legue *et al.*, 2014; Xu *et al.*, 2015; Yordanov *et al.*, 2017; Li *et al.*, 2018; Cai *et al.*, 2019; Wei *et al.*, 2020; Yue *et al.*, 2020; Zhang *et al.*, 2020; Xu *et al.*, 2021). Nevertheless, most research has so far focused on successive AR development stages in a given genotype; there have been no comparisons between easy-to-root and difficult-to-root genotypes.

To understand the underlying causes of poor-rooting and good-rooting in different genotypes, we compared the hybrid poplar clone OP42, which is easily propagated from dormant stem cuttings, and the hybrid aspen clone T89, which is unable to develop ARs under the same conditions.

Previous research has revealed that, predictably, ARs form from specific founder cells in poplar stem cuttings, but that the process is highly dependent upon induction treatment and age of the cutting (Rigal *et al.*, 2012). Cambium cells have also been shown to be competent initiators of ARs in *Eucalyptus* or *Populus* (Chiatante *et al.*, 2010; Chao *et al.*, 2019). Transcriptomic profiling of vascular tissues including the cambium region in *Populus* have been reported in several studies (Schrader *et al.*, 2004; de Almeida *et al.*, 2015; Kim *et al.*, 2019), but little attention has been given to gene expression in *Populus* cambial cells during AR development. Rigal *et al.* (2012) showed that changes in the transcriptome occur in the cambium during the early stages of AR development in *Populus*. In our present study we performed a global comparative transcriptomic analysis of the cambium of cuttings taken from OP42 and T89 clones.

Interestingly, the juvenile plants from the two clones rooted similarly when grown *in vitro* (Fig. 1). In both cases the ARs originate from the cambium region (Fig. 1). But the hybrid aspen T89, unlike the hybrid poplar OP42, was unable to develop roots from 3-month-old plants grown in the greenhouse (Fig. 2). Aging is a well-known limiting factor for AR development (reviewed in Diaz-Sala *et al.*, 2002; Bellini *et al.*, 2014; Aumond *et al.*, 2017) and this could be one explanation to the different behaviours observed between plants grown *in vitro* and those grown in the greenhouse for 3 months.

Interestingly, among the differentially expressed TFs, we found that the *P. trichocarpa PtHox52* gene (Potri.014G103000) was down-regulated in the cambium of the easy-to-root genotype OP42, and up-regulated in the difficult-to-root genotype T89, compared with OP42 at T_1_. This is surprising, since the *P. ussuriensis PuHox52* gene product has been described as a positive regulator of adventitious rooting in *P. ussuriensis* (Wei *et al.*, 2020). It was shown to induce nine regulatory hubs, including the JA signalling pathway driven by the *PuMYC2* gene (MH644082; Potri.002G176900), which was confirmed to be a positive regulator of AR development in *P. ussuriensis*. In contrast, JA signalling appears to be up-regulated in the cambium of the difficult-to-root T89 genotype compared with OP42, and we confirmed that *PtMYC2.1* negatively controls AR development in the hybrid aspen T89 (Fig. 6), as we had previously shown in Arabidopsis (Gutierrez *et al.*, 2012; Lakehal *et al.*, 2020a). These are intriguing results, but the role of JA in the control of AR development is still unclear, and seems to be context- and species-dependent (Lakehal *et al.*, 2020b). It will be interesting in the future to study whether *Populus MYC2* paralogues have acquired different functions depending on the species, growth and vegetative propagation conditions. Although T89 and OP42 clones rooted similarly *in vitro*, T89 was more sensitive to exogenously applied JA (Fig. 6). This result suggests that the higher up-regulation of the JA pathway in the cambium of T89 24 h after cutting could contribute to repress adventitious root initiation.

Interestingly, the orthologues of the three Arabidopsis *ARF* genes that were shown to be either positive (*AtARF6*, *AtARF8*) or negative (*AtARF17*) regulators of ARI in Arabidopsis (Gutierrez *et al.*, 2009, 2012; Lakehal *et al.*, 2019) behaved similarly in both T89 and OP42 (Fig. S6). An exception is *PttARF17.1*, which was significantly less expressed in the cambium of the difficult-to-root T89 compared with OP42 at both time points T_0_ and T_1_. This result agrees with a potential positive role of *PttARF17.1* in ARI, as described for *PeARF17* in the hybrid poplar *P. davidiana × P. bolleana* (Liu *et al.*, 2020). Nevertheless, down-regulation of *PttARF17.1* and *PttARF17.2* expression in T89 induced ARI (Fig. 5E), suggesting a negative role for *PttARF17.* As in Arabidopsis (Gutierrez *et al.*, 2009), when the expression of one of the three *PttARFs* was perturbed, the expression of the others was modified (Fig. 4). In this study, when down-regulation of *PttARF17* occurred, *PttARF6* paralogues were up-regulated, which probably contributed to increase ARI (Fig. 4E). As for *MYC2* genes, it is possible that different paralogues of *ARF17* have different functions, depending on the species or the context. We also observed that, as in Arabidopsis (Gutierrez *et al.*, 2009), *PttARF6*, *PttARF8* and *PttARF17* are likely to regulate the expression of one another at the transcriptional and post-transcriptional level through the microRNA pathway, suggesting that at least part of the regulatory mechanisms is conserved.

There were many TFs that were either up- or down-regulated in OP42 at T_1_ compared with T_0_, but not in T89, and their further characterization may certainly further advance our ­understanding of the mechanisms differentiating difficult-to-root from easy-to-root genotypes. In particular, several genes from the *LBD*, *NAC* and *GRAS* families of TFs, involved in root or vascular development, were found more specifically differentially expressed in OP42. Whether these genes account for the difference between the two genotypes requires additional functional characterization.

Another interesting difference we observed between T89 and OP42 concerns the expression of genes encoding ROS scavenging proteins. We identified 43 of these genes among the DEGs, 33 of which belong to the GST super-family, and 10 to the PEROXIDASE superfamily. The most striking observation was that 32 were significantly up-regulated in OP42 compared with T89 at T_1_, and 21 of those were also up-regulated in OP42 at T_0_ (Supplementary Fig. S5). Recent studies have shown that peroxidase activity positively regulates AR formation in different species (reviewed in Nag *et al.*, 2013; Li *et al.*, 2017; Velada *et al.*, 2018). It is therefore possible that the up-regulation of most of these genes in the cambium of OP42 compared with T89 partially explains the difference in rooting competence.

In conclusion, the comparison of the transcriptomes of the cambium region from two *Populus* species with opposite adventitious root phenotypes, showed a higher number of DEGs in the easy-to-root genotype compared with the difficult-to-root genotype. In particular, there were three times as many differentially expressed transcription factors in the easy-to-root genotype, several of which are known to be involved in adventitious root development, but many for which the function still needs to be addressed. Further functional characterization will shed light on their role in the differential competence to develop adventitious roots.

## Supplementary data

The following supplementary data are available at *JXB* online.

Fig. S1. Conditions for adventitious rooting assays from *in vitro* plants and greenhouse-grown plants.

Fig. S2. Workflow for laser capture microdissection (LCMS) of cambium tissues from stem cuttings.

Fig. S3. Quality assessment of the RNAseq data in the different biological replicates.

Fig. S4. *Populus* Arabidopsis orthologues of *ARF6*, *ARF8* and *ARF17* and their expression pattern in wood-forming tissues.

Fig. S5. Heat map showing the average expression of genes encoding ROS scavenging proteins in the cambium of T89 and OP42 genotypes.

Fig. S6. Heat map showing the average expression of *PtrARF* genes in the cambium of T89 and OP42 genotypes.

Fig. S7. Overexpression of *PtAF6.4* and PtARF8.2 under the control of the 35S promoter.

Fig. S8. Effect of exogenous auxin on the development of adventitious roots on T89 and OP42 cuttings.

Table S1. Primer list used in the present study.

Dataset S1. RNA-seq raw data:

Dataset S2. Lists of differentially expressed genes.

Dataset S3. Gene Ontology and list of differentially expressed transcription factors.

erac126_suppl_supplementary_dataset_S1

erac126_suppl_supplementary_dataset_S2

erac126_suppl_supplementary_dataset_S3

erac126_suppl_supplementary_figures_S1-S8_table_S1

## Data Availability

The RNA-seq data have been deposited at the European Nucleotide Archive (http://www.ebi.ac.uk/ena/) under the accession number PRJEB21558. RNA-seq data for OP42 and T89 can be accessed with the accession numbers PRJEB21549 and PRJEB21557, respectively).
