# Supplementary material for: Molecular basis of differential adventitious rooting competence in poplar genotypes
Source: J Exp Bot. 2022 Mar 24;73(12):4046–64. doi: 10.1093/jxb/erac126 (PMC9232201; doi:10.1093/jxb/erac126)
Supplement: erac126_suppl_supplementary_figures_S1-S8_table_S1 [file erac126_suppl_supplementary_figures_s1-s8_table_s1.pdf]

Molecular basis of differential adventitious rooting competence in poplar genotypes

Alok Ranjan[1,∮#], Irene Perrone[1,2#], Sanaria Alallaq[1,3#], Rajesh Singh[4†], Adeline Rigal[5], Federica Brunoni[1§], Walter Chitarra[2,6], Frederic Guinet[5], Annegret Kohler[5], Francis Martin[5], Nathaniel Street[1], Rishikesh Bhalerao[4], Valérie Legué[5∮], and Catherine Bellini[1,7,*].

The following Supporting Information is available for this article:

Supplementary Fig. S1 Conditions for adventitious rooting assays from *in vitro* plants and greenhouse-grown plants

Supplementary Fig. S2: Workflow for laser capture microdissection (LCMS) of cambium tissues from stem **cuttings**

Supplementary Fig. S3: Quality assessment of the RNAseq data in the different biological replicates

Supplementary Fig. S4: Populus Arabidopsis orthologues of *ARF6*, *ARF8* and *ARF17* and their expression pattern in wood-forming tissues

Supplementary Fig. S5: Heat map showing the average expression of genes encoding ROS scavenging proteins in the cambium of T89 and OP42 genotypes

Supplementary Fig. S6: Heat map showing the average expression of *PtrARF* genes in the cambium of T89 and OP42 genotypes

Supplementary Fig. S7: Over-expression of *PtAF6.4* and PtARF8.2 under the 35S promoter

Supplementary Fig. S8: Effect of exogenous auxin on the development of adventitious roots on T89 and OP42 cuttings

Supplementary Table S1 Primer list used in the present study.

Supplementary Dataset S1 (excel document):

Sheet 1 Library-size-normalised variance-stabilised data set

Sheet 2 Expression values for the 17,997 expressed genes

Supplementary Dataset S2: Differentially expressed genes

Sheet 1 The differentially expressed genes (DEG) up- and down-regulated and their annotation.

Sheet 2 Total number of DEG in OP42 when compared at time T1 and T0.

Sheet 3 The DEG up-regulated in OP42 at time T1 compared to time T0.

Sheet 4 The DEG down-regulated in OP42 at time T1 compared to time T0.

Sheet 5 Total number of DEG in T89 when compared at time T1 and T0.
Sheet 6 The DEG up-regulated in T89 at time T1 compared to time T0.
Sheet 7 The DEG down-regulated in T89 at time T1 compared to time T0.
Sheet 8 The total number of DEG in OP42 and T89 at time T0.
Sheet 9 The number of DEG up-regulated in T89 compared to OP42 at timeT0.
Sheet 10 The number of DEG down-regulated in T89 compared to OP42 at timeT0.
Sheet 11 Total number of DEG between T89 and OP48 at time T1.
Sheet 12 The number DEG up-regulated in T89 compared to OP42 at time T1.
Sheet 13 The number of DEG down-regulated in T89 compared to OP42 at time T1.

Supplementary Dataset S3

Sheet 1 Vascular tissue expressed genes set.
Sheet 2 Differentially expressed Transcription Factors.
Sheet 3 Differentially expressed ROS scavenging proteins
Sheet 4 Gene Ontology of up-regulated DEGs.
Sheet 5 Gene Ontology of down-regulated DEGs.
Sheet 6 Mean of expression values used for the heat maps

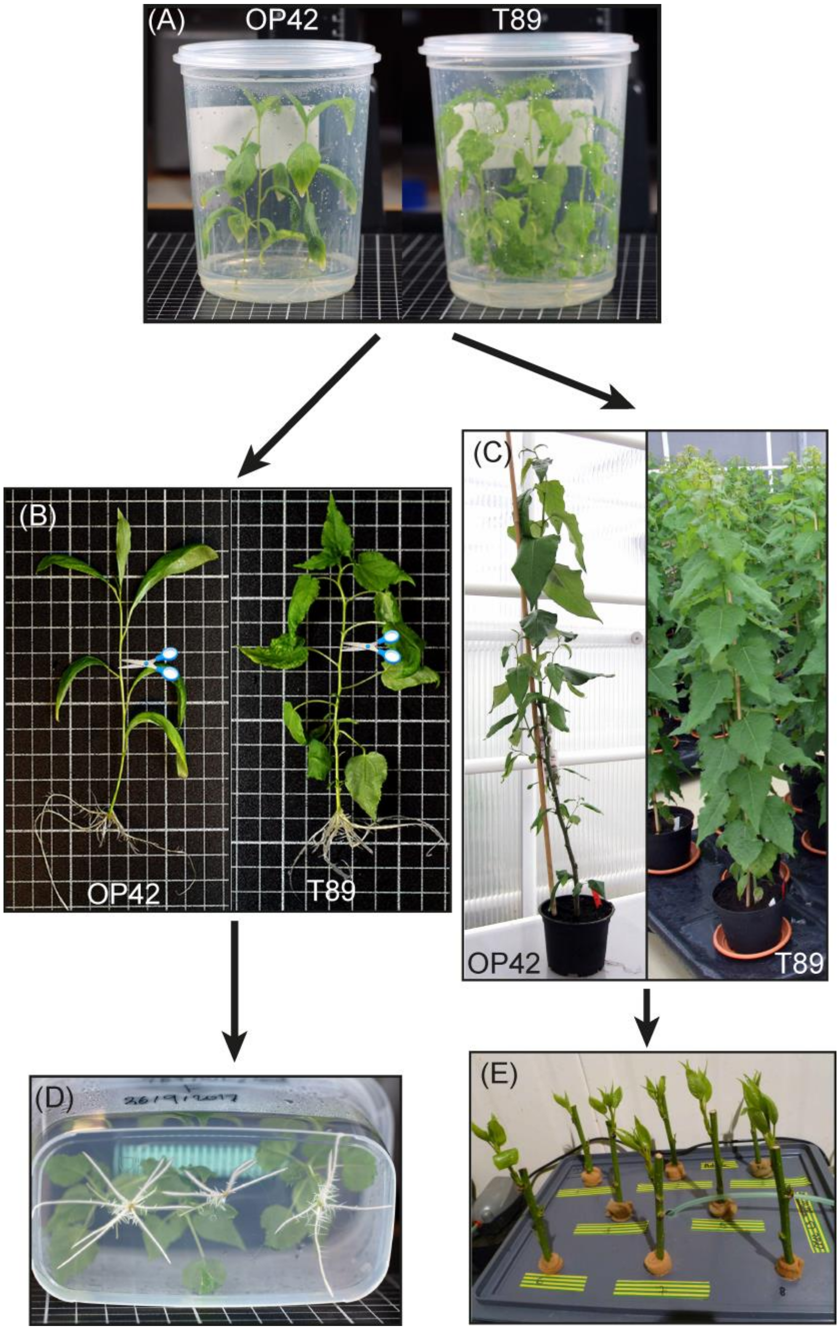

Fig. S1: Conditions for adventitious rooting assays from *in vitro* plants and greenhouse-grown plants

(A) OP42 and T89 plants are propagated under *in vitro* conditions for four weeks.

(B, D) Cuttings comprising the shoot apex and the three first internodes starting from the shoot apex were excised from 4-week-old plants (B) and transferred to fresh ½ MS medium in smaller rectangular jars (D). The number of AR was monitored, starting 5 days after being cut, when the

first macroscopic events could be observed at the base of the cuttings, until 14 days after cutting as in (D).

(C) Four-week-old *in vitro* OP42 and T89 plants were transferred into pots containing soil and left to grow for three months in the greenhouse. (E) Approximately 20 cm stem cuttings with a 1 cm stem diameter were excised from the three-month-old plants and transferred in hydroponic conditions.

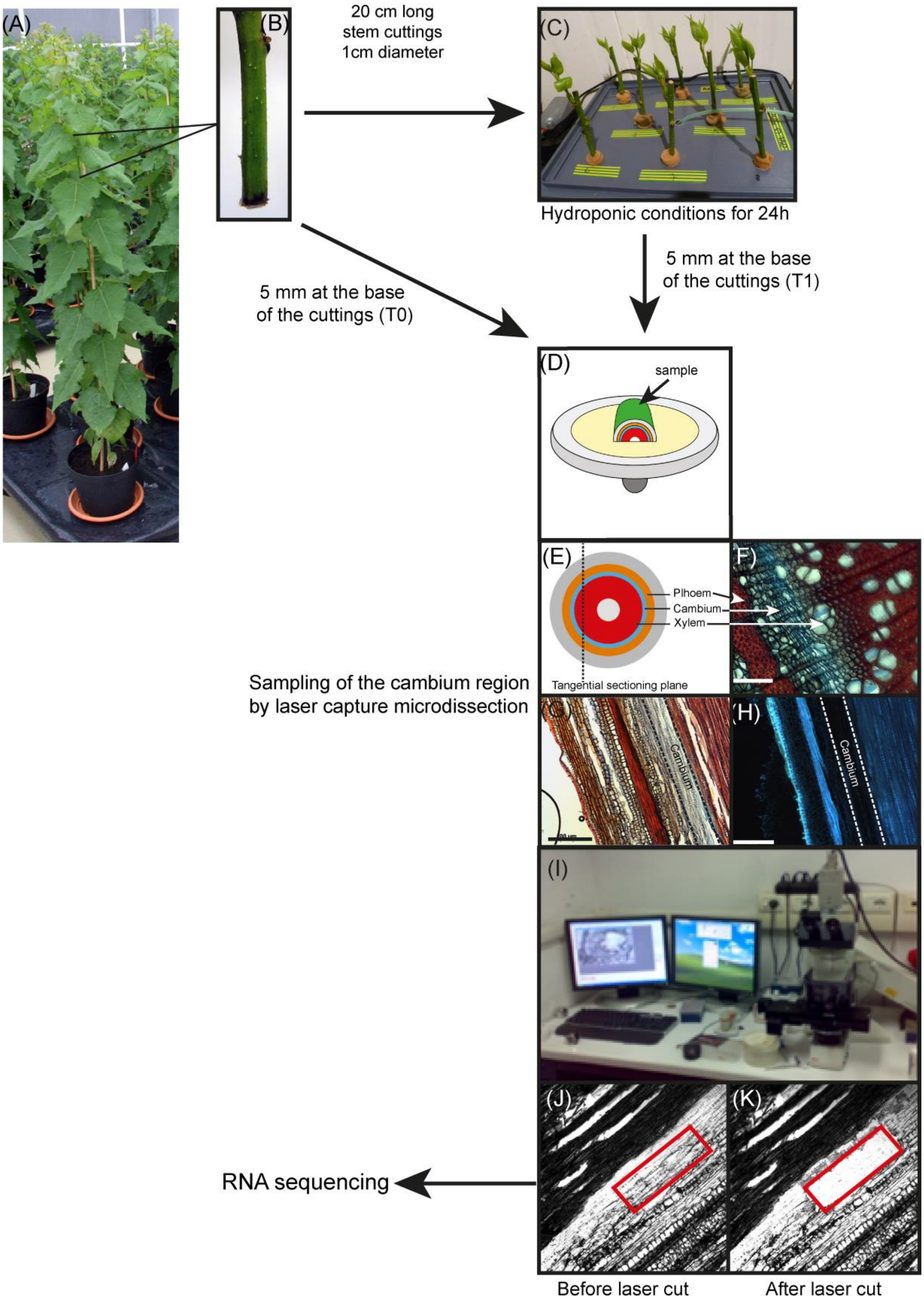

(A)
(B)
20 cm long
stem cuttings
1cm diameter
(C)
Hydroponic conditions for 24h
5 mm at the base
of the cuttings (T1)
5 mm at the base
of the cuttings (T0)
(D)
sample
(E)
Plhoem
Cambium
Xylem
Tangential sectioning plane
F)
Sampling of the cambium region
by laser capture microdissection
Cambium
(H)
Cambium
(I)
(J)
(K)
RNA sequencing
Before laser cut
After laser cut

Fig. S2: Workflow for laser capture microdissection (LCMS) of cambium tissues from stem cuttings

(A) T89 and OP42 plants were grown in the greenhouse for 3 months.

(B) 20 cm lengths of stem cuttings were taken as for the hydroponic assay (Supplementary Figure 1) and 5 mm long pieces were cut at their base, flash frozen in liquid nitrogen and used for cambium tissue sampling at time T0.

(C) A second set of stem cuttings were kept in hydroponic conditions for 24 h (T1) and 5 mm long stem pieces were cut at the base of the cuttings, flash frozen in liquid nitrogen and used for cambium tissue sampling at time T1.

(D) 5 mm stem pieces were split in half longitudinally.

(E) Schematics of the anatomy of a stem; (F) cross-section of a stem cutting; (G and H) Longitudinal cryosection of the base of a stem cutting observed under the microscope of the micromanipulator with white light (G) or UV light which allowed us to identify more precisely the cambium region which did not show any fluorescence (H). (I) computerised system for LCMS. Cambium region before (J) and after (K) laser microdissection.

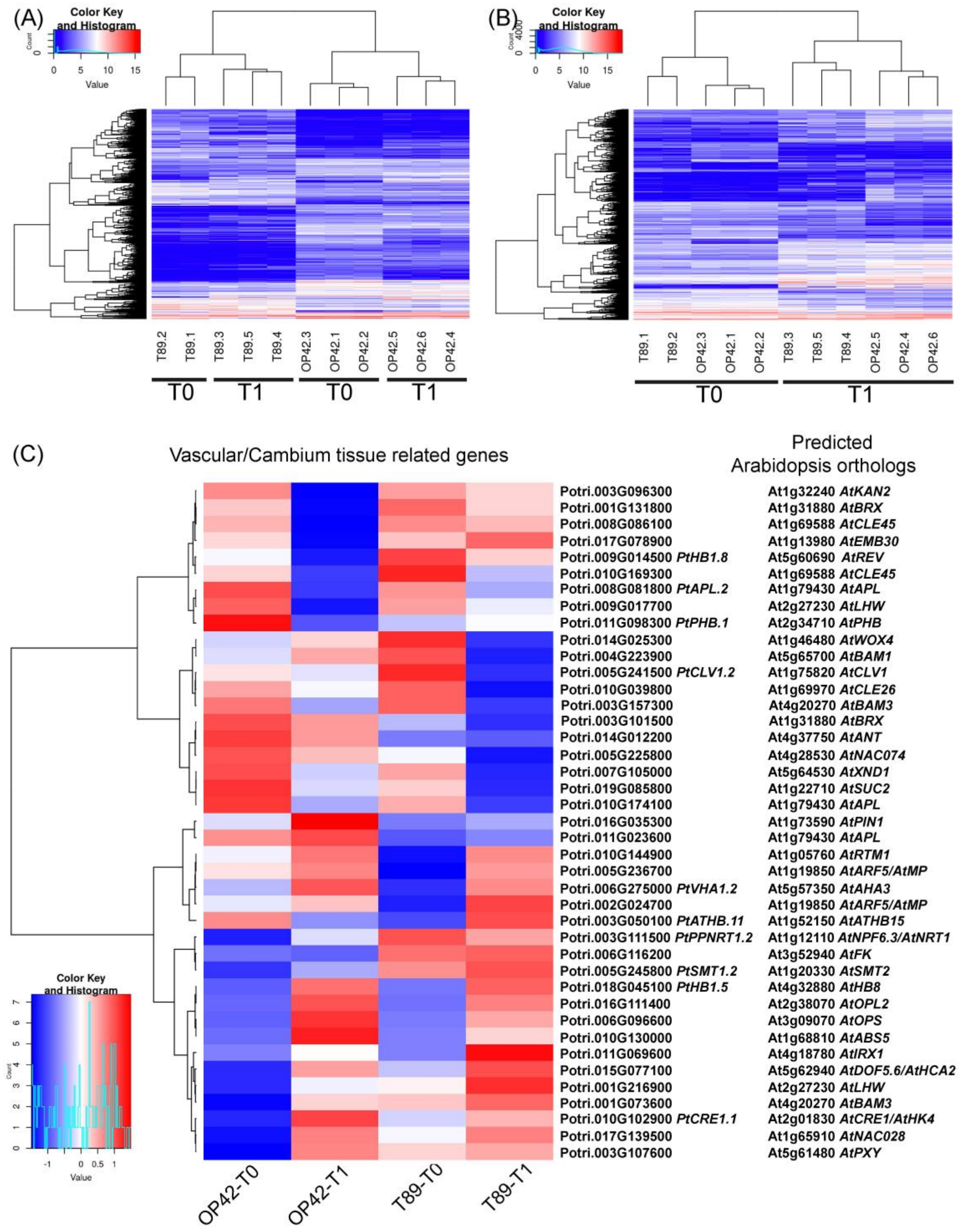

Fig. S3: Quality assessment of the RNAseq data in the different biological replicates

(A) The dendrogram of samples (top) was divided into two parts based on the correlation between a genotype's gene expression and then labelled (bottom), respectively. (B) The dendrogram of

samples (top) was divided into two parts based on the correlation between time and a treatment's gene expression and then labeled (bottom), respectively. (C) The heat map was generated based on genotypes (T89 and OP42) and time after cutting. T0 immediately after cutting, and T1 24 h after cutting and being transferred to hydroponic conditions. Heatmaps of DE genes (DE cut-offs of FDR ≤ 0.01 and $|LFC| \geq 0.5$), were generated using the function heatmap.2 from the gplots R library. The genes, which were expressed in either one or two biological replicates, but which expression was significantly different between T89 and OP42, were also mapped with the variance stabilising transform (VST) data set. The gene expression mean values used for the heat map are listed in Supplementary data set 3, sheet 6.

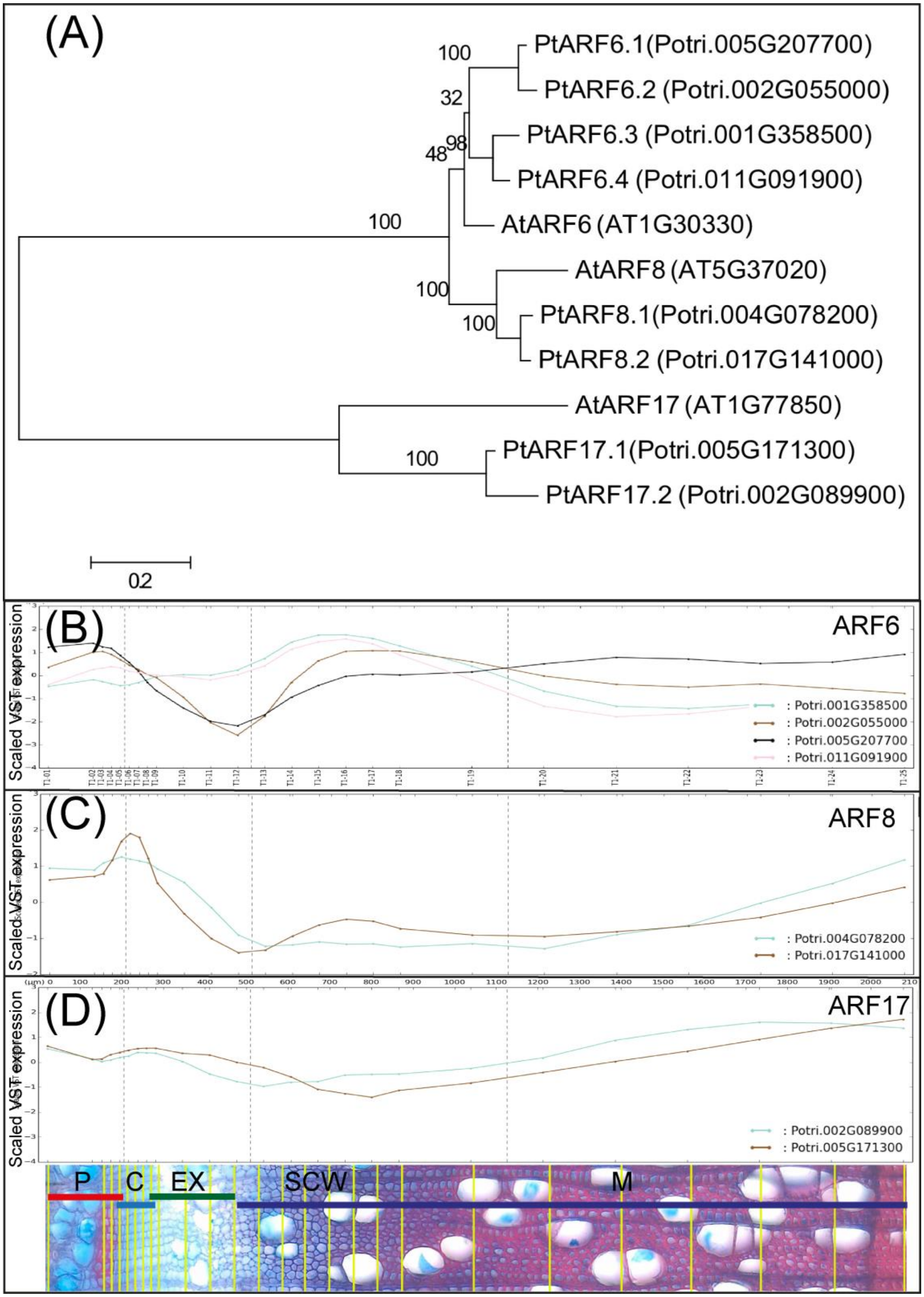
(A)
PtARF6.1(Potri.005G207700)
PtARF6.2 (Potri.002G055000)
PtARF6.3 (Potri.001G358500)
PtARF6.4 (Potri.011G091900)
AtARF6 (AT1G30330)
AtARF8 (AT5G37020)
PtARF8.1(Potri.004G078200)
PtARF8.2 (Potri.017G141000)
AtARF17 (AT1G77850)
PtARF17.1(Potri.005G171300)
PtARF17.2 (Potri.002G089900)
02
(B)
ARF6
Scaled VST expression
: Potri.001G358500
: Potri.002G055000
: Potri.005G207700
: Potri.011G091900
(C)
ARF8
: Potri.004G078200
: Potri.017G141000
(D)
ARF17
: Potri.002G089900
: Potri.005G171300
P
C
EX
SCW
M

Fig. S4: **Populus Arabidopsis orthologues of ARF6, ARF8 and ARF17 and their expression pattern in wood-forming tissues**

(A) Phylogenetic relationship between *P. trichocarpa* and *Arabidopsis thaliana* ARF6, ARF8 and ARF17 proteins. Protein sequences were aligned with ClustalW and the phylogenetic analysis was performed in Mega 8 using the Neighbour-Joining method with a bootstrap test (1000 replicates). (B-D) Expression patterns of *ARF6*, *ARF8* and *ARF17* genes in the wood-forming regions of aspen trees (http://aspwood.popgenie.org). The y-axis shows the variance-scaled expression. The x-axis shows tangential samples over the wood-developing tissues with four zones indicated: P = phloem; C + EX = cambium and expansion zones; SCW = secondary cell wall deposition zone; M = maturation zone (Sundell *et al.*, 2017). The corresponding Potri. identifications are *PtARF6.1*, Potri.005G207700; *PtARF6.2*, Potri.002G055000; *PtARF6.3*, Potri. 001G358500; *PtARF6.4*, Potri.011G091900; *PtARF8.1*, Potri.004G078200; *PtARF8.2*, Potri.017G141000; *PtARF17.1*, Potri.005G171300; *PtARF17.2*, Potri.002G089900.

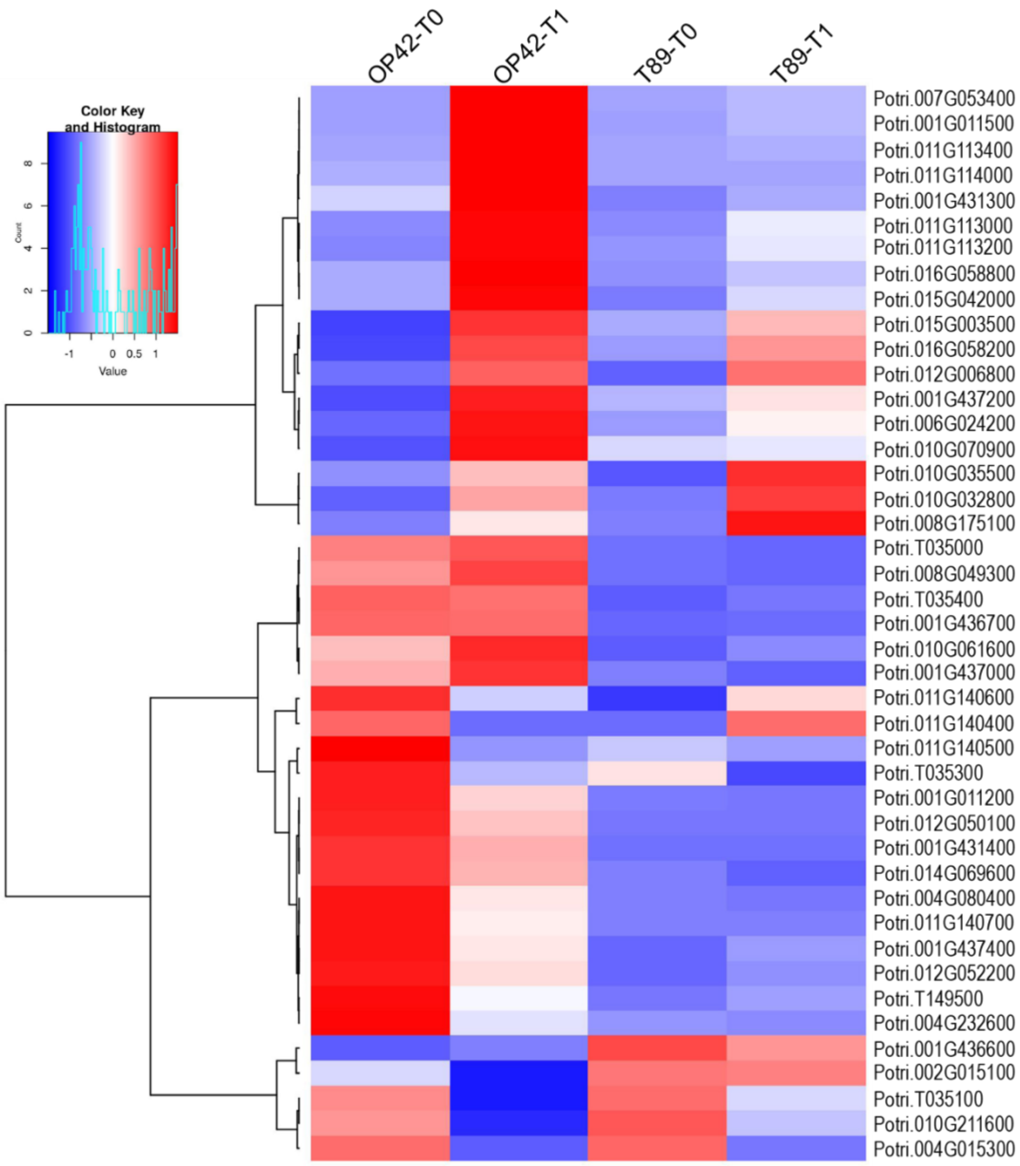

Fig. S5: Heat map showing the average expression of genes encoding ROS scavenging proteins in the cambium of T89 and OP42 genotypes

The heat map was generated based on genotypes (T89 and OP42) and time after cutting. T0 immediately after cutting and T1 24 h after cutting and being transferred to hydroponic conditions. (Supplementary data sets 2 and 3, sheets 3 and 4). The heat map was generated based on genotypes (T89 and OP42) and time after cutting. T0 immediately after cutting and T1 24 h after cutting and being transferred to hydroponic conditions. Heat maps of DE genes (DE cut-offs of FDR ≤ 0.01 and |LFC| ≥ 0.5), were generated using the function heatmap.2 from the gplots R library. The

genes, which were expressed in either one or two biological replicates, but which expression was significantly different between T89 and OP42, were also mapped with the variance stabilising transform (VST) data set. The gene expression mean values used for the heat map are listed in Supplementary data set 3, sheet 6.

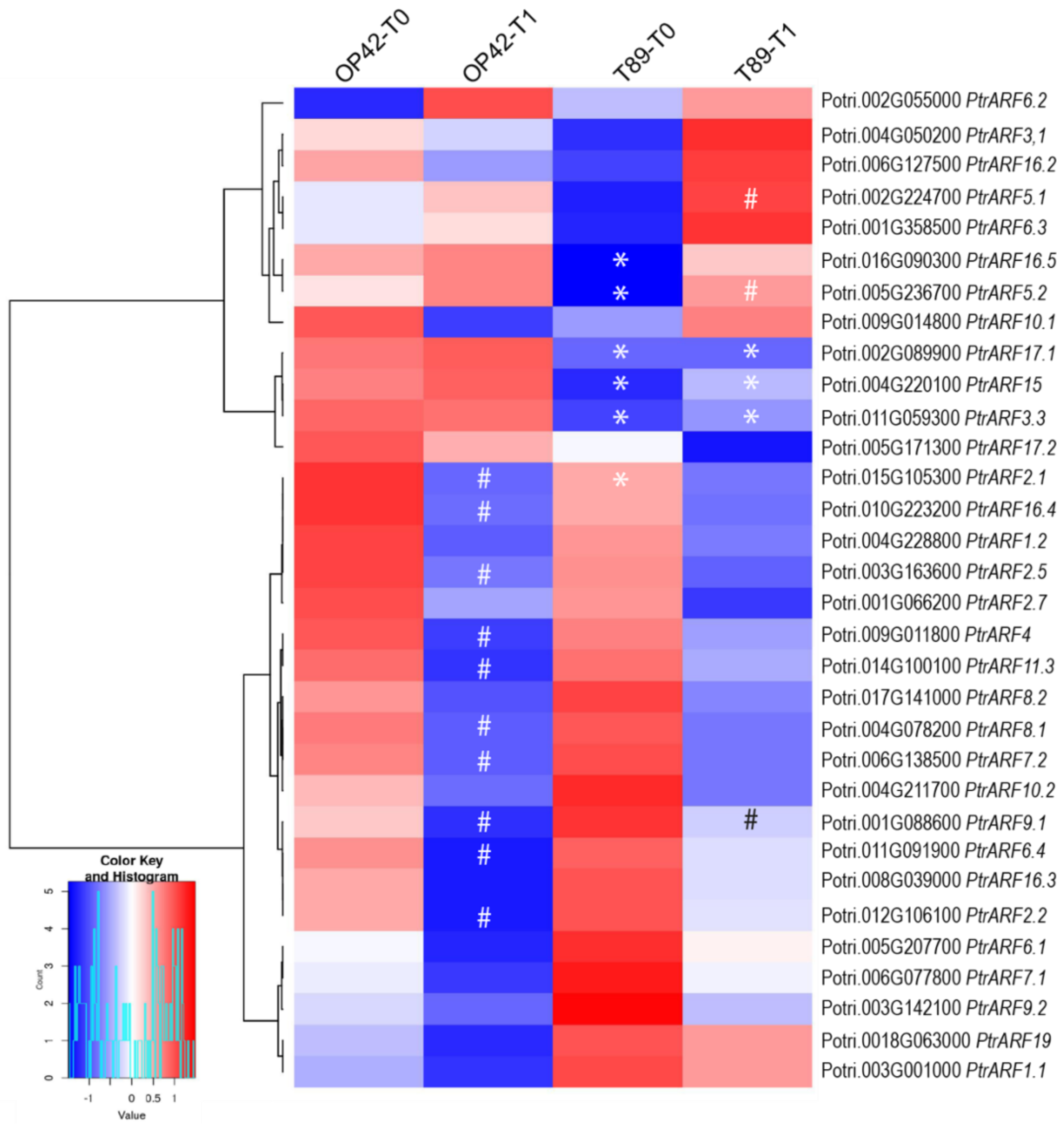

Fig. S6: Heat map showing the average expression of *PtARF* genes in the cambium of T89 and OP42 genotypes

The heat map was generated based on genotypes (T89 and OP42) and time after cutting. T0 immediately after cutting and T1 24 h after cutting and being transferred to hydroponic conditions. The asterisks indicate that the expression in T89 compared to OP42 at T0 or T1 is significantly different. The dashes indicate that the expression at T1 compared to T0 in either T89 or Op42 is significantly different (Supplementary data sets 2 and 3). The gene expression mean values used for the heat map are listed in Supplementary data set 3, sheet 6.

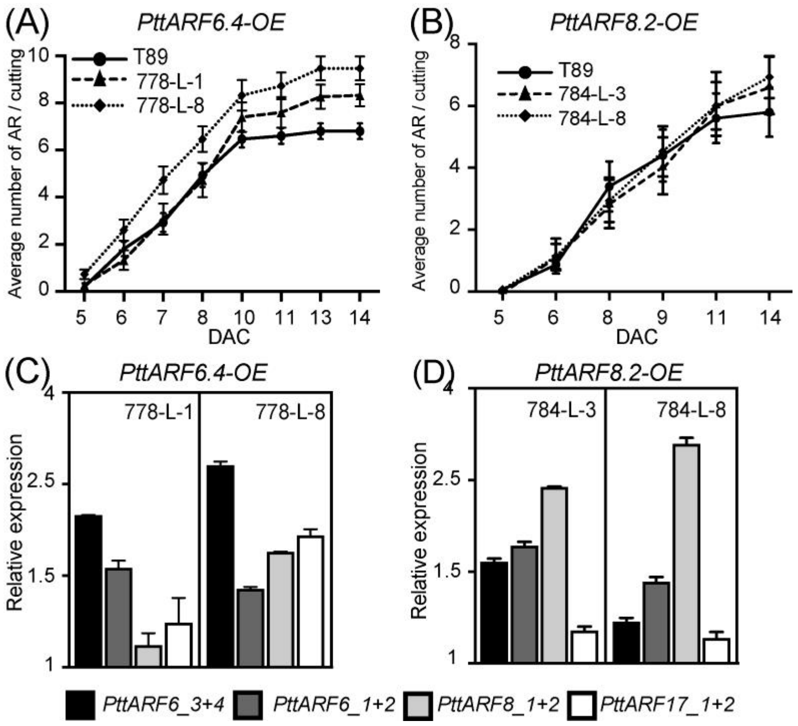

Fig. S7: Over-expression of *PtAF6.4* and PtARF8.2 under the 35S promoter

(A-B) Average number of AR on cuttings of transgenic plants expressing *p35S:PtARF6.4* (A) and *p35S*:*PtARF8.2* (B) Rooting assays were performed as described in Materials and Methods. Two independent transgenic lines were compared to the control T89. AR number was scored every day starting day 5 after being cut until 14 days after cutting (DAC). For each line 15 cuttings were analysed. Data are means ± SE, n = 15, corresponding to two independent lines per construct.

(C-D) The *PtARF6.1/2*, *PtARF6.3/4*, *PtARF8.1/2*, *PtARF17.1/2* un-cleaved transcript abundance was quantified in stem cutting fragments of *p35S:PtARF6.4* and *p35S*:*PtARF8.2* over-expressing lines and the control line T89. Gene expression values are relative to the reference gene and calibrated towards the expression in the control line T89, for which the value is set to 1. Error bars indicate SE obtained from three independent biological replicates. A one-way analysis of variance combined with the Dunnett's comparison post-test indicated that the values marked with an asterisk were significantly different from T89 values ($P < 0.05$; $n = 3$).

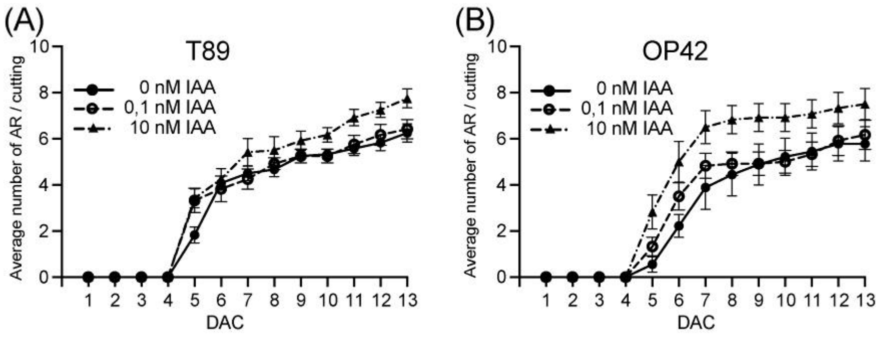

Fig. S8: Effect of exogenous auxin on the development of adventitious roots on T89 and OP42 cuttings

Average number of AR in stem cuttings of (A) T89 and (B) OP42 in the absence or presence of 0.1 nM or 10 nM of IAA (indole acetic acid). For each line and each condition 12 cuttings were analysed.

Data are means ± SE, n = 12. Three independent biological replicates were used.

A two-way ANOVA with a Tukey's multiple comparisons test indicated that a concentration of 10 nM of IAA induced adventitious rooting in a similar way in both genotypes.

Table S1 Primer list used in the present study.

		Forward Primer	Reverse Primer
	**Cloning Primers**		
Potri.011G091900	PtARF6.4	CACCATGAGGCACTCTTCGGCTTC	TTAAATTTCTCGGCAGTCCAAAGAC
Potri.017G141000	PtARF8.2	CACCATGAAGCTTTCAACATCAGG	TCATCCTTTGACAGCATTTGGGCC
Potri.001G358500/ Potri.011G091900	PtARF6.3/4 RNAi	CACCACTGCTGCGTTTCAGGAGAT	ATGAGATGTTTCGTCCTGGG
Potri.004G078200/ Potri.017G141000	PtARF8.1/2RNAi	CACCCAAATTTCAACAGAAAGCTTGC	GTAGATTGACCAGCTCTGGAGA
Potri.005G171300/ Potri.002G089900	PtARF17.1/2RNAi	CACCAACGGTGGTGGTTTCTCCGTC	ACCGCCACCAGCAATCTGCT
Potri.003G092200	PtMYC2	CACCATGACTGATTACCGTCTA	CTATCGGGCATCACCAACTTTTGT
	**qPCR Primers**		
Potri.001G358500/ Potri.011G091900	PtARF6.3/4	GAGTTGCGAAGTGAGCTTGC	TTACAAATTCCGGCCAGGGG
Potri.005G207700/ Potri.002G055000	PtARF6.1/2	ATGATGAGCTTCGCAGTGAGC	AGGATCATCACCAAGGAGAAGC
Potri.004G078200/ Potri.017G141000	PtARF8.1/2	GGACATATCCCGGTTCAGCA	ACTCCCAGGGATCATCTCCAA
Potri.005G171300/ Potri.002G089900	PtARF17.1/2	CCCAATGAAGAAATTGAGATATCC	GAATGTGGAAAAAGGATCTTGC
Potri.003G092200	PtMYC2.1	CTACGAGCTGTGGTTCCTAATGTAT	ATTTGACATCTTAAGCTCCTGATTG
Potri.001G418500	PtUBQ	GTTGATTTTTGCTGGGAAGC	GATCTTGGCCTTCACGTTGT
